# High-throughput screening data generation, scoring and FAIRification: a case study on nanomaterials

**DOI:** 10.1186/s13321-025-01001-8

**Published:** 2025-04-23

**Authors:** Gergana Tancheva, Vesa Hongisto, Konrad Patyra, Luchesar Iliev, Nikolay Kochev, Penny Nymark, Pekka Kohonen, Nina Jeliazkova, Roland Grafström

**Affiliations:** 1 Ideaconsult Ltd., 4 Angel Kanchev Str, 1000 Sofia, Bulgaria; 2 Department of Analytical Chemistry and Computer Chemistry, Faculty of Chemistry, University of Plovdiv, 24 Tsar Assen St, 4000 Plovdiv, Bulgaria; 3 Division of Toxicology, Misvik Biology, Karjakatu 35 B, 20520 Turku, Finland; 4 Institute of Environmental Medicine, Karolinska Institutet, 171 77 Stockholm, Sweden

**Keywords:** NAMs, Nanomaterials, HTS, eNanoMapper, ToxPi, FAIR, Nanomaterials grouping, Automatic workflow

## Abstract

In vitro-based high-throughput screening (HTS) technology is applicable to hazard-based ranking and grouping of diverse agents, including nanomaterials (NMs). We present a standardized HTS-derived human cell-based testing protocol which combines the analysis of five assays into a broad toxic mode-of-action-based hazard value, termed Tox5-score. The overall protocol includes automated data FAIRification, preprocessing and score calculation. A newly developed Python module ToxFAIRy can be used independently or within an Orange Data Mining workflow that has custom widgets for fine-tuning, included in the custom-developed Orange add-on Orange3-ToxFAIRy. The created data-handling workflow has the advantage of facilitated conversion of the FAIR HTS data into the NeXus format, capable of integrating all data and metadata into a single file and multidimensional matrix amenable to interactive visualizations and selection of data subsets. The resulting FAIR HTS data includes both raw and interpreted data (scores) in machine-readable formats distributable as data archive, including into the eNanoMapper database and Nanosafety Data Interface. We overall present a HTS-driven FAIRifed computational assessment tool for hazard analysis of multiple agents simultaneously, including with broad potential applicability across diverse scientific communities.

**Scientific Contribution** Our study represents significant tool development for analyzing multiple materials hazards rapidly and simultaneously, aligning with regulatory recommendations and addressing industry needs. The innovative integration of in vitro-based toxicity scoring with automated data preprocessing within FAIRification workflows enhances the applicability of HTS-derived data application in the materials development community. The protocols described increase the effectiveness of materials toxicity testing and mode-of-action research by offering an alternative to manual data processing, enrichment of HTS data with metadata, refining testing methodologies—such as for bioactivity-based grouping—and overall, demonstrates the value of reusing existing data.

## Introduction

The development of new chemical substances and advanced materials, including NMs, pose complex challenges to ensure safety for humans and the environment. “New Approach Methodologies” (NAMs) refers to scientific techniques and approaches for laboratory safety assessments without animal testing under the 3Rs principle [1]. NAMs encompass innovative in vitro- and in silico-driven technologies, including high-throughput screening with human cell culture models. Stakeholders promoting the application of NAMs to safety assessments include regulatory agencies and industry. For the latter, the evaluation of safety overlaps innovation, and safe and sustainable by design manufacturing [2–5].

Data management based on FAIR [6] (Findability, Accessibility, Interoperability, and Reuse) guiding principles support consistent machine-driven curation and reuse of the accumulated data by the nanosafety, cheminformatics and bioinformatics communities. Tools supporting FAIRification of safety data are available, i.e., the Nanosafety Data Interface [7] provides flexible findability of data gathered and/or generated by a wide variety of European nanosafety projects. The data entry for newly generated data is streamlined through the eNanoMapper Template Wizard [8], which facilitates efficient processing of widely accepted excel data format by means of a user-friendly online form that allows users to specify essential experimental information for which they require data and associated metadata. The data entry is further supported by the newly developed Template Designer online app that automates the step 2 of the protocol published in [8] (creating custom data entry excel templates). The eNanoMapper FAIRification workflow [9] is applied to convert data entered into harmonized templates to the eNanoMapper data model [7, 9] and to import results into the database or generate NeXus files.

The complexity of reproducible assessment and simultaneous validation of many agent effects makes application of HTS technology challenging for defining adversity effects. Implementing FAIR principles in the context of HTS data presents further challenges, including automatically linking large experimental data sets to descriptive metadata, harmonizing the terminology used, converting them into a machine-readable format that will allow the data to be found, accessed and reused. Traditional HTS results documentation approaches, such as using spreadsheets for data collecting and preprocessing are time-consuming and error-prone. Alongside the challenges associated with manual data processing, integrating external software like ToxPi [10, 11] into the workflow introduces further complexity, especially due to the need of transferring substantial data sets. While ToxPi serves as a valuable tool for data visualization and harmonization, its capabilities are constrained by the absence of preprocessing functions and limited output options.

Herein we report a plate-based toxicity assessment procedure utilizing stand-alone plate-replicators, -fillers and -readers for assessment of five commonly used toxicity endpoints under multiple timepoints, concentrations, replicates, and cell models. We couple additionally the HTS analysis with a set of automated FAIRification protocols that improve results readability and enhance the interpretation of results in diverse scientific settings.

## Results

**Fig. 1:**
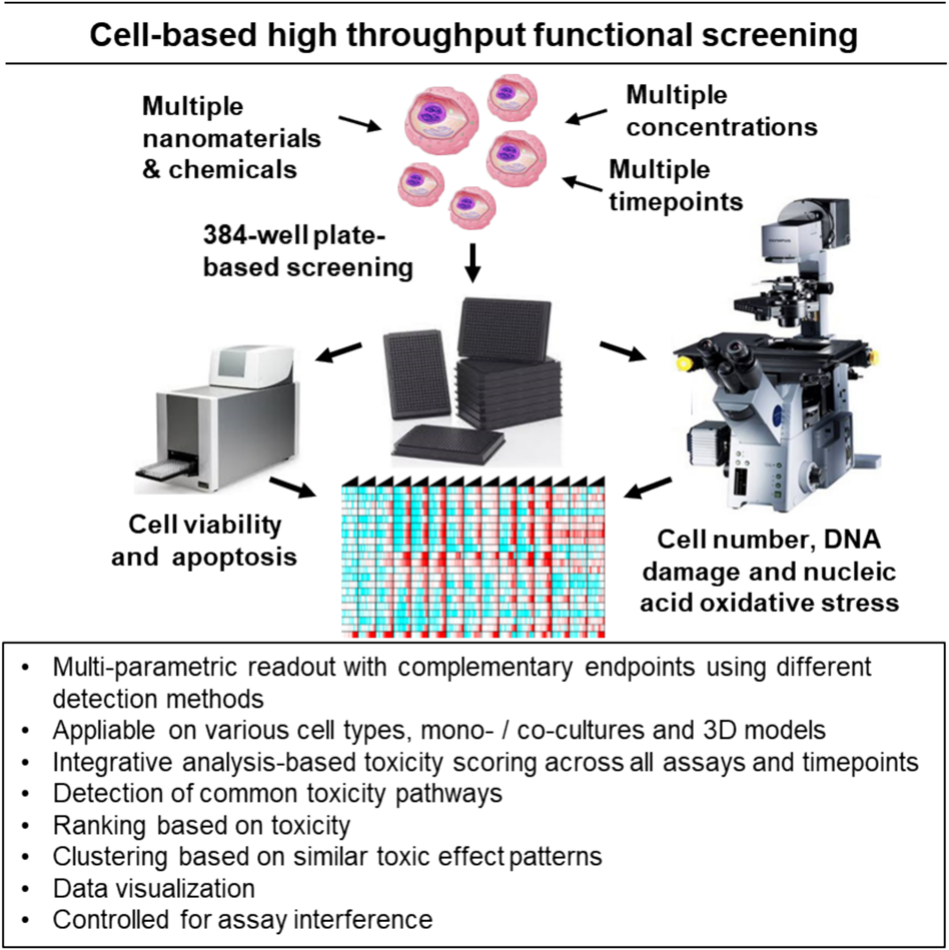
Cell based high throughput functional screening by Misvik Biology

The HTS set-up builds on an in-house developed next-generation hazard assessment workflow which applies high-throughput and high-content profiling technologies integrated with omics profiling for assessing toxicity of chemicals and NMs (previously described [5, 12, 13]). The tiered approach to NMs safety evaluation is initiated by HTS, where a panel of toxicity tests are applied for relative toxic potency ranking of multiple agents (Fig. 1). The Misvik high throughput screening setup enables rapid toxicity assessment of multiple materials using a set of well-established toxicity endpoints (CellTiter-GloⓇ for cell viability, DAPI for cell number, gammaH2AX for DNA damage, 8OHG for nucleic acid oxidative stress, and Caspase-GloⓇ 3/7 for apoptosis), and several time points, adapted from [12, 13].

The use of multiple exposure times provides a kinetic dimension to the test and combination of luminescence and fluorescence-based endpoints generate complementary readouts that control for potential assay interference by the tested agents. In the second step gene expression profiling and toxic mode of action screening is carried out to gain a deeper understanding of the toxicity mechanisms involved.

The approach developed is demonstrated using two different HTS datasets from the H2020 HARMLESS [14] and calibrate [15] EU projects. The datasets consists of 30 NMs, 5 reference chemicals and one nanomaterial control, which were assessed for toxicity using 5 in vitro hazard assays, three time points, and two human cell models.

The caLIBRAte data encompass a HTS-based evaluation of a total of 28 NMs, along with five selected chemical controls and one nanomaterial control acting as reference points for in vitro hazard assessments. Table 1 summarizes caLIBRAte assays, which include five toxicity assays with a minimum of three time points were carried out in BEAS-2B cells assayed in the presence and absence of 10% serum in the culture medium. Four biological replicate screens using a twelve-concentration dilutions series for each material were carried out. Number of data points obtained is indicated.

**Table 1:** Summary of assays carried out for the caLIBRAte project

Endpoint	Assay (unit)	Mechanism	Time points (h)	Concentration points	Biological replicates	Data points
Cell viability *Luminescence measurement*	CellTiter-Glo assay (RLU)	ATP metabolism	0, 6, 24, 72	12	4	12 288
Cell number *Imaging*	DAPI staining (cell number)	DNA content	6, 24, 72	12	4	18 432
Apoptosis *Imaging*	Caspase-3 activation (RFI)	Caspase-3 dependent apoptosis	6, 24, 72	12	4	9216
Nucleic acid oxidative damage *Imaging*	8OHG staining (RFI)	Oxidative stress	6, 24, 72	12	4	9216
DNA double-strand breaks *Imaging*	γH2AX staining (RFI)	DNA repair	6, 24, 72	12	4	9216
Total						58 368

Data for quantum dots, from the HARMLESS project, were used to test and demonstrate flexibility of the developed workflow. Deviations from the caLIBRAte materials and methods are described in section Methods.

**Fig. 2:**
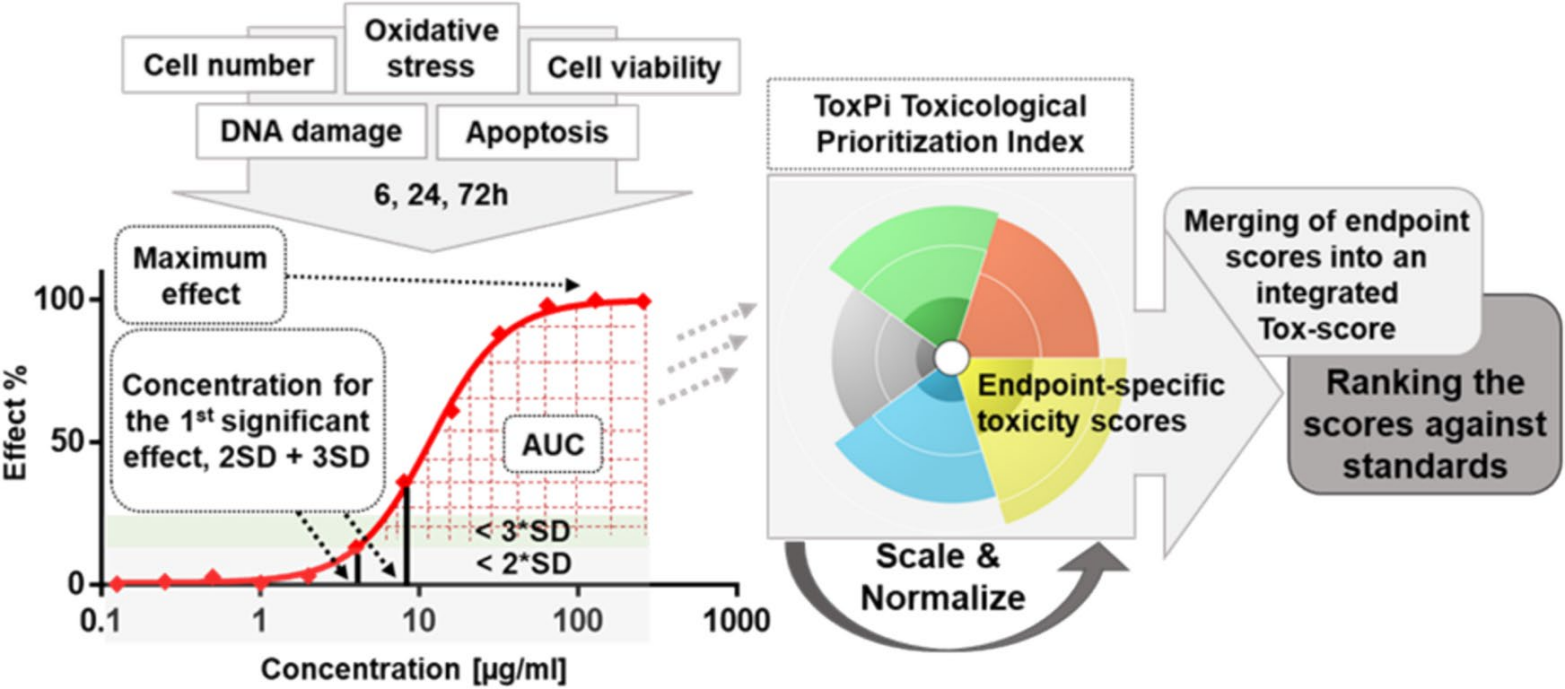
Tox5-scoring and ranking of the in-vitro nanomaterial toxicity dose–response data

Traditional toxicity testing is based on determination of the growth inhibitory 50 (GI_50_) value based on which sequential assays are carried out. Combining toxicity results from several time points and endpoints (e.g., induction of Apoptosis and γH2AX) may provide more sensitive and specific toxicity estimates. However, GI_50_ cannot be calculated or is not optimal for some of the endpoints used here. Thus, a GI_50_–independent scoring system was used (Fig. 2). Steps taken from the traditional one-endpoint, one-time point 50% growth inhibition concept to a more comprehensive, multi-time and -endpoint concept, enabling toxicity scoring. Toxicity is measured using several time points and multiple complementary endpoints and key metrics (1st statistically significant effect, AUC and maximum effect) are calculated from the data. The metrics are separately scaled and normalized using the ToxPi software to allow for comparability, and then compiled to end- and time-point-specific toxicity scores, which are further compiled to an integrated Tox5-score, which is used as the basis for toxicity ranking and grouping against well-known toxins.

The concept is based on calculation of multiple metrics from the normalized dose–response data and integration of those metrics from several time points and endpoints into one integrated toxicity score, which retains transparency towards the contribution of each specific endpoint.

Tox5-score [16] integrates dose–response parameters from different endpoints and conditions (time points, cell lines, concentrations) into a final toxicity score. Each slice shows the bioactivity and weight of each specific endpoint. The pie serves as a basis for computational assessment of similarity in toxicity responses, i.e. toxicity profiles. The complexity and visualization of the pie may vary depending on the amount of information included. The transparency of the approach allows a clear visualization of the overall assessment, allowing chemicals and materials to be ranked from most to least toxic and comparison with known chemical toxicants included in the screens. Furthermore, the new workflow enables the combination of data from other experiments with similar setups, enhancing its versatility and applicability. Clustering, based on endpoint, timepoint and cell line specific toxicity scores, enables grouping and read-across, including transparency on the grouping hypothesis, i.e. the underlying bioactivity associated with the detected hazard-based similarity.

### General concept for automated HTS data preprocessing and Tox5-score approach

**Fig. 3:**
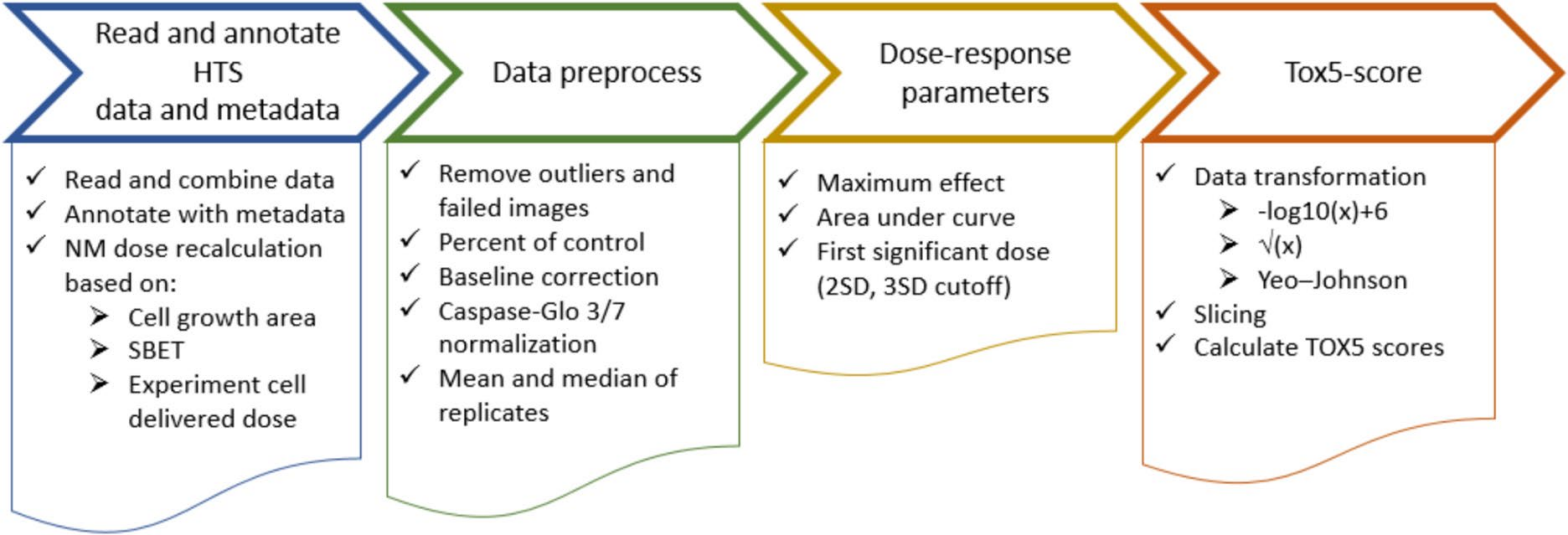
General steps in the workflow for HTS data preprocessing and toxicity scoring

To allow for automated evaluation of the toxicity of chemicals and materials, and allow for grouping and ranking using the Tox5-score approach, a general computational workflow was developed, as presented in Fig. 3. This general concept can be implemented using various computational techniques within the frame of MS Excel or other calculation software.

#### Reading experimental HTS data and metadata annotation

First, experimental data are read, combined, and converted into a uniform format suitable for post-processing. Metadata, including details such as concentration, treatment time, type of material used, cell line, and replicate, are also provided and used for annotation.

Here, it is worth noting that when investigating toxicity of non-soluble (nano)materials, the cell-delivered doses play a crucial role. Traditionally, toxicity evaluation is based on *nominal* dose, i.e., the mass of chemical/material per volume unit of cell culture medium, but as (nano)material sedimentation rates, hydrophobicity and other physical chemical factors influence the interaction between cell culture medium and cells, the fraction of material mass actually reaching the cells remains unknown. Consequently, quantification of material delivered to close proximity of the cells is crucial, as material situated far from the cells does not contribute to toxicity, except in the case of material dissolution. Additionally, a NMs toxicity can be directly linked to its surface area [17]—generally, more surface area implies more toxicity. Utilizing Brunauer–Emmett–Teller (BET) or Sears titration analysis, the concentrations can be expressed as material surface area per cell growth area (cm^2^/cm^2^) concentration units, which can further enhance the scoring process. It's important to note that the concentrations in points 1 and 2 [nominal dose per cell culture medium (μg/ml) and nominal dose per cell growth area (μg/cm^2^)] can be computationally determined. However, for point 3 [nominal dose based on the material’s specific surface area per cell growth area (cm^2^/cm^2^)], empirical Specific Surface Area, measured by BET method (SBET), is necessary to determine the material’s specific surface area, which is then used to calculate the concentration. To take material’s surface area information and cell delivered dose into account, a dose recalculation was incorporated to the initial step of the workflow, enabling selection of concentration units between:

1. nominal dose per cell culture medium (μg/ml),
2. nominal dose per cell cell growth area (μg/cm^2^),
3. nominal dose based on the material’s specific surface area per cell growth area (cm^2^/cm^2^), and
4. the cell delivered dose per cell growth area (cell delivered cm^2^/cm^2^).

caLIBRAte project data are presented in units of μg/ml and can be recalculated as μg/cm^2^ based on cell growth area, and HARMLESS data can be expressed using any of the above mentioned concentration units as results for SBET are available. BET and Sears correlation based surface area information and cell delivered dosing was acquired by the HARMLESS project partners.

#### HTS data preprocessing and dose–response parameters

The second step of the workflow involves data preprocessing to minimize the impact of systematic errors caused by human, biological, or technical factors during the experimental process, leading to unwanted variation and noise in the data. The data were expressed as percent of plate-wise controls (percent of control) to account for plate-to-plate and screen-to-screen variability. Since percent of control is sensitive to outliers in the controls, outlier removal was applied to control samples by defining the 75th (Q3) and the 25th (Q1) percentiles and their interquartile range (IQR). Control samples with values greater than Q3+ 1.5*IQR or less than Q1−1.5*IQR were removed. Additionally, a 0-h exposure served as a baseline for cell viability assays, representing the pre-exposure state and also indicating possible assay interference. A baseline correction was applied by subtracting the median of replicate 0-h baseline values from the corresponding time-point replicates.

In-vitro imaging endpoints have been detected by fluorescence microscopy from the same wells. Cells have been detected through nuclear DAPI staining, and the absence of signal could be attributed to either absence of cells or failed image focusing (e.g., focus on a dust particle instead of the cells), resulting in two distinct methods for cleaning DNA imaging data. In both scenarios, no 8OHG and H2AX signals were obtained leading to NaN (Not a Number) values for H2AX and 8OHG. When cells have been absent, the cell count has been set to 0 if biological replicates showed cell counts close to zero, preserving meaningful information, i.e., all cells have been most likely dead as there were few cells in the replicates. DAPI values have been further refined by replacing 0 with NaN when the mean DAPI values of replicates have exceeded a threshold of 50. This strategy eliminated DAPI values that resulted from focusing errors or other technical issues. The same algorithm has been applied for Caspase-3 normalization using a second technical replicate for DAPI signal from the same well. For the DAPI signal, the average of the two technical replicates has been used.

Since Caspase-Glo 3/7 activation (measured as a viability assay in HARMLESS data) has been used as a sign of dying cells, the Caspase readout, in addition to 0-h assay interference subtraction and normalization to plate-wise controls, has been further normalized to cell number. This has been achieved by dividing the Caspase readout by the mean of the CTG and DAPI responses to account for the loss of cells.

To minimize the influence of outliers, median values of the biological replicates have been calculated and used for determining the dose–response parameters in the third step.

#### Tox5-score computation

Maximum effects were calculated separately for each replicate, and median maximum effect values were used for scoring. For Area under the curve (AUC) calculations, values less than two standard deviations (2SD) from the median of vehicle-treated controls were excluded to remove baseline noise. AUC calculations were performed using a non-curve fitting-based trapezoid rule, and values beyond the highest concentration x-values were not extrapolated. The first significant effect defines the concentration threshold at which a statistically significant response is induced compared to control conditions. Only significant effect values higher than the median of controls plus two standard deviations (2SD) and plus 3SD were considered to exclude artifacts. Two separate metrics for + 2SD and + 3SD cutoffs were calculated. The two cut-offs were included so that the scoring extended to the lower toxicity materials/agents while still giving higher weight to the higher toxicity ones. Likewise the maximum effect calculations help rank materials at the lowest toxicities that fall below even the 2SD cut-off.

The final part of the workflow involves toxicity scoring and grouping of the materials. The ToxPi v2.1 software [18] has been used to scale, normalize and integrate time point-, and endpoint-specific metrics. Toxicological effects from the material library were first scaled between 0 and 1, and transformed to smooth the data towards normal distribution. This was done separately for each time- and end-point and dose response parameter so that 1st significant effect data were − log10(x) + 6 transformed, and AUC and maximum effect data were square root transformed.

These dose–response parameter scores were combined into slices to generate endpoint and time point specific composite scores, which constituted the final pie representing the integrated Tox5-score.

To emphasize sensitivity of the observed toxicity effects, the first significant effect doses (with 3SD cutoff) were weighed in the final slices so that they contributed to 50% of the final score while other parameters contributed to 16.67% each. 95% confidence intervals were calculated for the integrated Tox5-scores, rank numbers, and endpoint- and time-point-specific scores. Due to the effect-thresholds applied for the AUC and 1st significant effect values, low toxicity materials get a toxicity score, which might be based only on the maximum effect values. Thus, the scoring and ranking accuracy declines as toxicity decreases, as less data is available for scoring, but a score is obtained even for the least toxic materials, as well as for the dispersant controls.

### Collection and annotation of HTS data

**Fig. 4:**
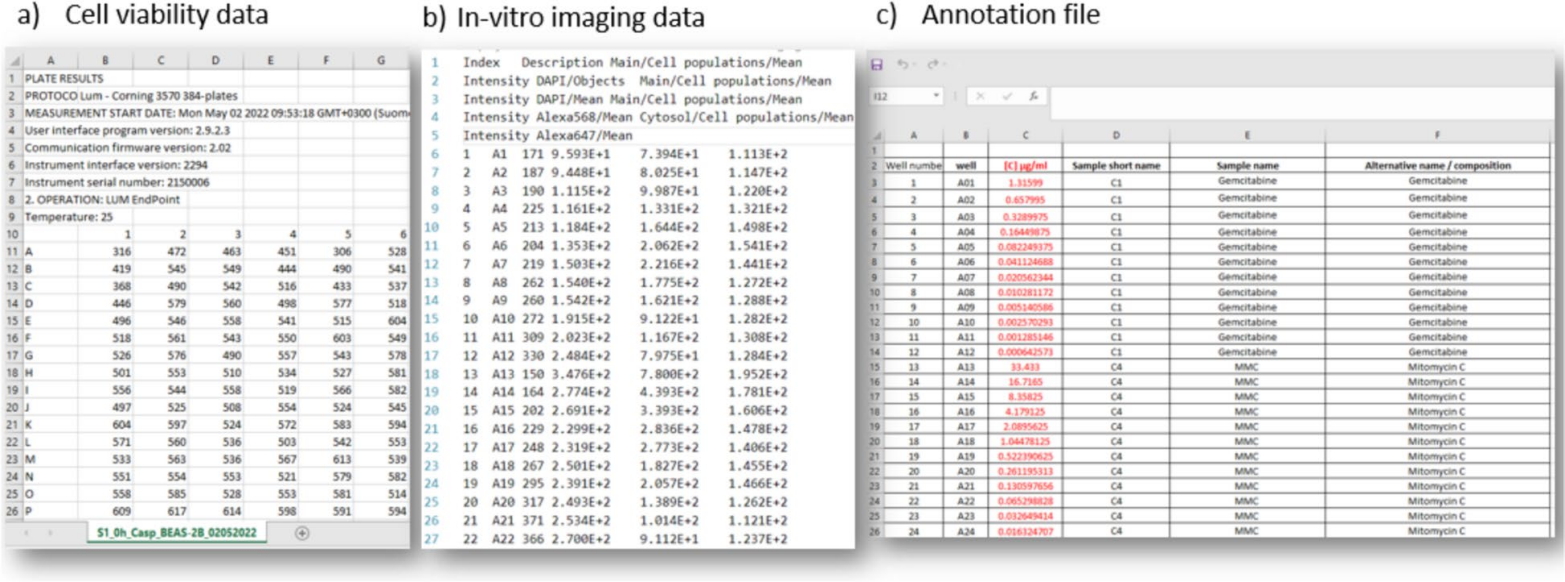
Screenshot of experimental data: **a** plate reader, **b** imaging data output files and **c** annotation file

In this study, in-vitro imaging data were provided as.txt files (Fig. 4b), while cell viability data (HARMLESS dataset) were in.csv format (Fig. 4a). Images were analyzed using Olympus ScanR software, with data exported as.txt files (see “Methods” section).

Each file corresponds to a 384-well plate, with cell viability data stored separately, and imaging data combined into one file. Metadata, such as well location, dose, time, replication, endpoint, and cell line, should be included with the raw data. An Excel annotation file (Fig. 4c) details the materials and doses in each well.

Collecting and annotating experimental metadata is challenging and time-consuming due to a lack of standardization, with inconsistent terminology across materials, endpoints, and cell lines (e.g., HepG2, HEPG2, HEP-G2). This issue complicates data FAIRification, emphasizing the need for automated machine-based data processing.

**Fig. 5:**
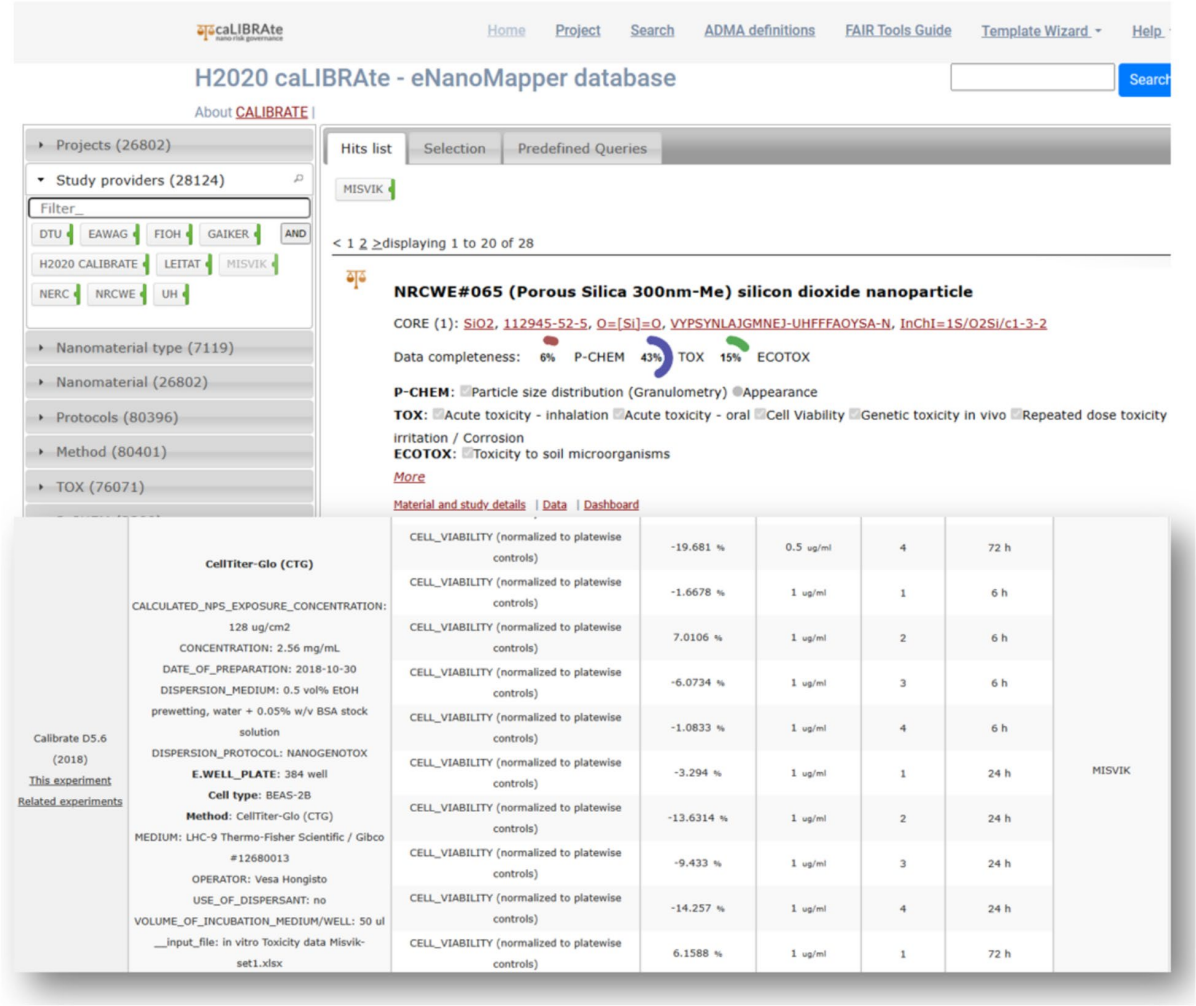
Screenshot of caLIBRAte cell viability data available in the eNanoMapper database at https://enanomapper.adma.ai/projects/calibrate

CellTiter-Glo assay data, from caLIBRAte project have been made available in the eNanoMapper database [7] (Fig. 5) and were managed in our previous activities. This process required manually crafting numerous JavaScript Object Notation (JSON) file configurations and adapting to changes in the layout of the input Excel files.

The HTS_METADATA template [19] as a harmonized reporting format is specifically designed, as part of this research, and integrated in Template Wizard [8] web form into Nanosafety Data Interface [7].

**Fig. 6:**
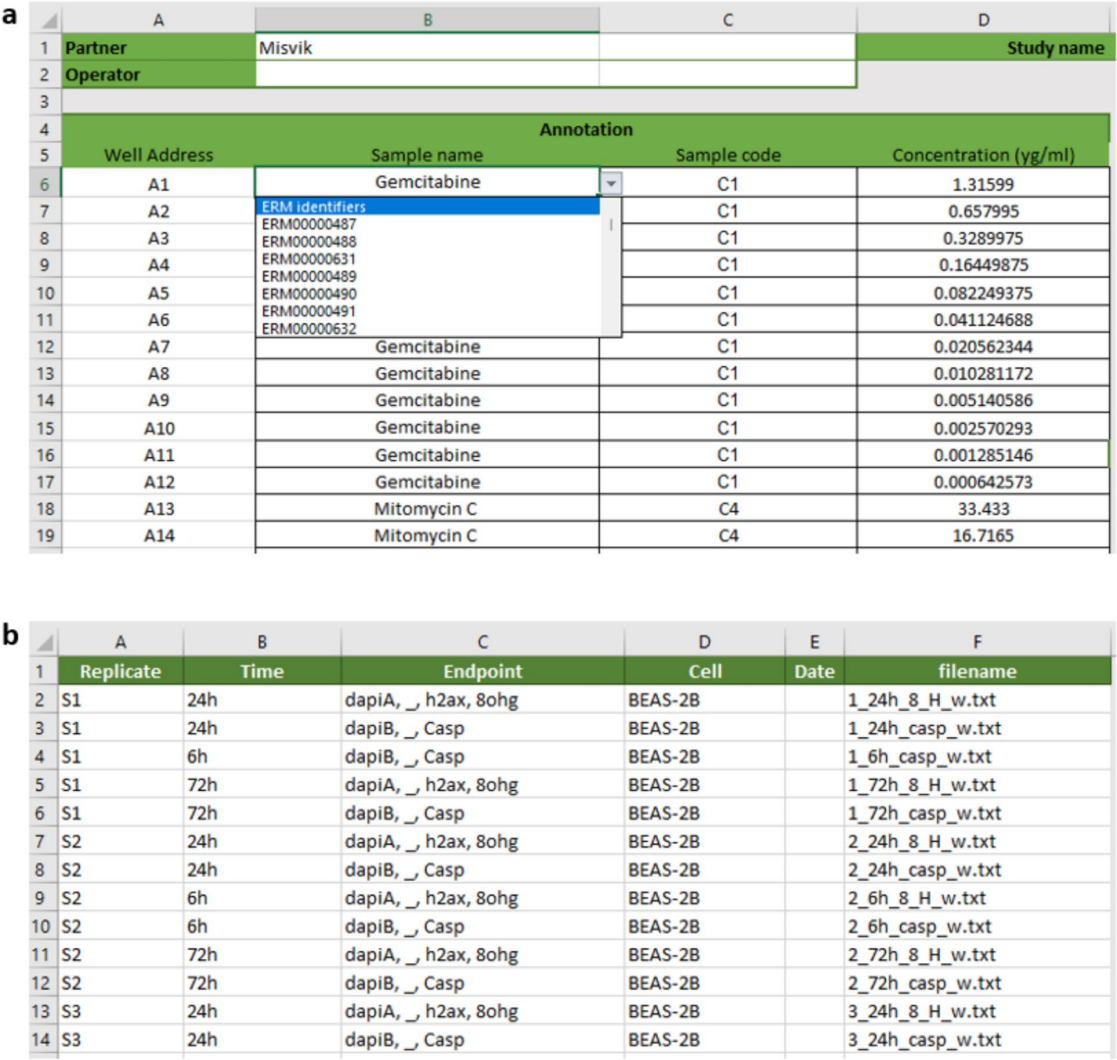
HTS_METADATA template: Example of filled template for Misvik HTS data. **a** Front sheet, **b** files sheet

The harmonized HTS metadata template includes a “Front sheet” (Fig. 6a) for metadata about materials and doses for a given well position, and a “File” sheet (Fig. 6b) for replicate, time, endpoints, and cell lines, auto-filled from data filenames. The auto-completed filename metadata can be corrected manually, which will ensure that the different spellings of the objects are harmonized. Users can conveniently select materials, presented with ERM identifiers [20], from a drop-down menu, in “Front sheet”, associated with the “Material” sheet.

The HTS_METADATA template allow users to annotate data with meaningful metadata while ensuring consistency and harmonization.

### Implementation of general concept in ToxFAIRy python package

The ToxFAIRy Python software was designed to automate HTS data preprocessing and toxicity scoring. The software is available in the orange3-toxfairy GitHub repository https://github.com/ideaconsult/orange3-toxfairy. The software consists of two main modules: endpoints and calculations. The “endpoints” module reads the data, annotates it with metadata, and collects both raw and processed data within a dedicated data container for each specific endpoint. The “calculations” module implements the latter stages of the general workflow outlined previously. It encompasses functionalities for data preprocessing, calculations of dose–response parameters, and the generation of Tox5-scores. Notably, Tox5-scores are derived by integrating the ToxPi-R library [21] into the Python workflow.

#### Reading and annotating the data

A dedicated Python method was developed to automatically populate the “files” sheet in the HTS_METADATA template using file names. After manual updates, if needed, the template is used to parse raw data and annotate it with metadata, stored in a two-dimensional labeled structure. Each endpoint is a separate instance of an HTS object, containing raw and processed data along with associated metadata. Dose recalculation functions are based on the following formulae:

1 $$Cell{ - }delivevered\;dose \left( {\frac{\mu g}{{cm^{2} }}} \right) = \frac{{dose\left( {\frac{\mu g}{{ml}}} \right) \times well\;volume\left( {\mu l} \right)}}{{plate\;growth\;area\left( {cm^{2} } \right)}}$$

2 $$SBET\;effective\;dose\left( {\frac{{cm^{2} }}{{cm^{2} }}} \right) = SBET\left( {\frac{{cm^{2} }}{g}} \right) \times \frac{{\frac{{dose\left( {\frac{\mu g}{{ml}}} \right) \div 1000}}{{well\;volume\left( {\mu l} \right)}}}}{{plate\;growth\;area\left( {cm^{2} } \right)}}$$

#### Data preprocessing

The “calculations” module contains five Python classes: one for basic normalizations, which is applied to all endpoints, two classes for cell viability and in-vitro imaging data processing, which inherit the basic normalization, and two classes for dose–response and Tox5-score calculations.

##### Basic normalization

Representation of the result as a percentage of the control based on Eq. 3

3 $$\begin{gathered} \% \;effect\;of\;median\;control = \frac{Sample\left( i \right)}{{median\;control}}*100 \hfill \\ \% \;of\;median\;control = \left( {1 - \left( {\frac{Sample\left( i \right)}{{median\;control}}} \right)} \right)*100 \hfill \\ \end{gathered}$$

Both approaches provide insights into the sample’s relationship with the control, offering flexibility in how the data is interpreted and presented.

Outlier removal based on Inter Quartile Range (IQR) between 75th (Q3) and 25th (Q1) percentiles of the data and calculation of mean and median of replicates.

##### Cell viability normalization

For endpoints involving a 0-h experiment baseline correction functionalities have been implemented based on Eq. 4. The baseline-corrected values can also be represented as a percentage.

4 $$\begin{gathered} {\text{Baseline}}\;{\text{correction}} = {\text{Sample}}\;{\text{i}}/{\text{median}}\left( {0{\text{ H}}} \right) \hfill \\ {\text{Baseline}}\;{\text{correction}}\;{\text{as}}\;{\text{percent}} = 100 + \left( {\frac{{{\text{Sample}}\left( {\text{i}} \right)}}{{median\left( {0H} \right)}}} \right) \hfill \\ \end{gathered}$$

Endpoint (Caspase-Glo 3/7) specific normalization step was implemented (Eq. 5) based on the combined mean data from two additional endpoints (DAPI and CTG used in the current case), to account for the loss of cells.

5 $${\text{Casp - Glo }}3/7\;{\text{normalization}} = \frac{{{\text{Sample }}\left( {\text{i}} \right)}}{{1 - ({\text{average }}\left( {{\text{mean}}\;{\text{endpoint }}1,{\text{ mean}}\;{\text{endpoint }}2} \right) \div 100}}$$

##### In-vitro imaging normalization

ToxFAIRy implements functionalities to remove potentially failed imaging based on DAPI assay results. According to the rules, if DAPI is 0, associated imaging endpoints are replaced with 0 and if the median of DAPI’s replicates > 50, the result is replaced with NaN.

##### Dose response parameters

ToxFAIRy identifies of the first significant dose using a 2SD/3SD cutoff, defining the dose where a noticeable response is induced compared to control conditions. P-values are calculated for each replicate using a two-tailed hypothesis test, and if p < 0.05, the null hypothesis is rejected, indicating a significant effect. Before this, p-values are filtered if the mean score of replicates is less than the control median plus 2 or 3 standard deviations (SD). These values establish the threshold for the initial significant concentration.

To improve AUC reliability and reduce minor fluctuations, a data cleaning step is performed as described early: data points are excluded if the mean score for a replicate endpoint was less than the control median + 2SD as well as AUC is determined using the trapezoidal rule. A logarithmic transformation is applied to the doses before AUC calculation, to comply with the trapezoidal rule assumption that the x-values (dose levels) are normally distributed. Maximum dose calculation was implemented as a median of maximum results of all replicates.

#### Tox5-score

Tox5-score calculations are performed by the ToxPi-R library [21] integrated into the ToxFAIRy. To achieve a normal distribution of the data, three different transformation functions were implemented: − log10(x) + 6, square root, and the Yeo-Johnson transformation. In ToxFAIRy, slicing can be done both automatically and manually, unlike in ToxPi, where slices must be entered manually one at a time. Tox5-scoring in ToxFAIRy software is enhanced with a more flexible weighting functionality, allowing specific parameters within the same slice to be weighted—an option not available in the original ToxPi.

ToxFAIRy calculates bootstrap confidence intervals for each specific slice, general Tox5-score and ranks at a 95% confidence level, similar to the original ToxPi [22]. Therefore, the reported toxicity scores are accompanied by measures of statistical significance.

ToxFAIRy module implements functionality for hierarchical clustering, allowing users to select from various distance metrics, including: Euclidean, Cityblock, Cosine, Hamming, and Minkowski. The set of supported clustering methods includes agglomerative clustering (with Ward’s method, Single linkage, Complete linkage, Average linkage). The number of clusters can be user defined or using the Elbow and Silhouette methods to determine the optimal number of clusters. Additionally, three cluster significance metrics (Silhouette score, Davies–Bouldin score, and Calinski–Harabasz score) are included.

### Orange3-ToxFAIRy add-on

**Fig. 7:**
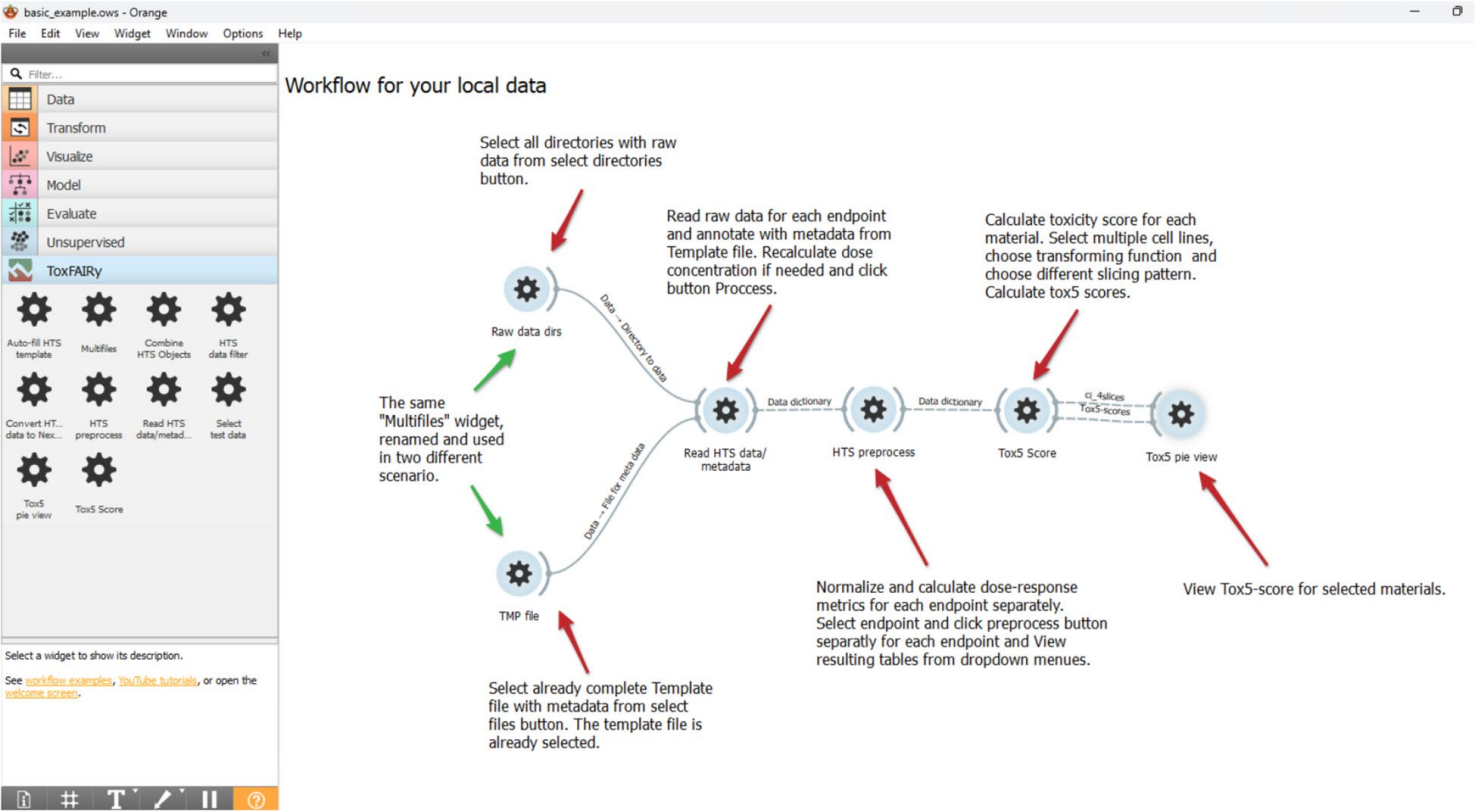
Basic example of an automated workflow for HTS data processing and Tox5-scoring

To facilitate HTS data preprocessing and scoring by non-programmers, we developed Orange3-ToxFAIRy, an add-on for the popular Orange Data Mining software. Orange3-ToxFAIRy wraps all ToxFAIRy functionalities in a user-friendly manner. The user can build a complete HTS data preprocessing and Tox5-score workflow (Fig. 7) by connecting custom widgets. (i.e. visual programming style). The workflows can be saved as.ows files and loaded later or shared as an informational resource. Orange3-ToxFAIRy visual user guide is available as a resource in Zenodo [23].

**Fig. 8:**
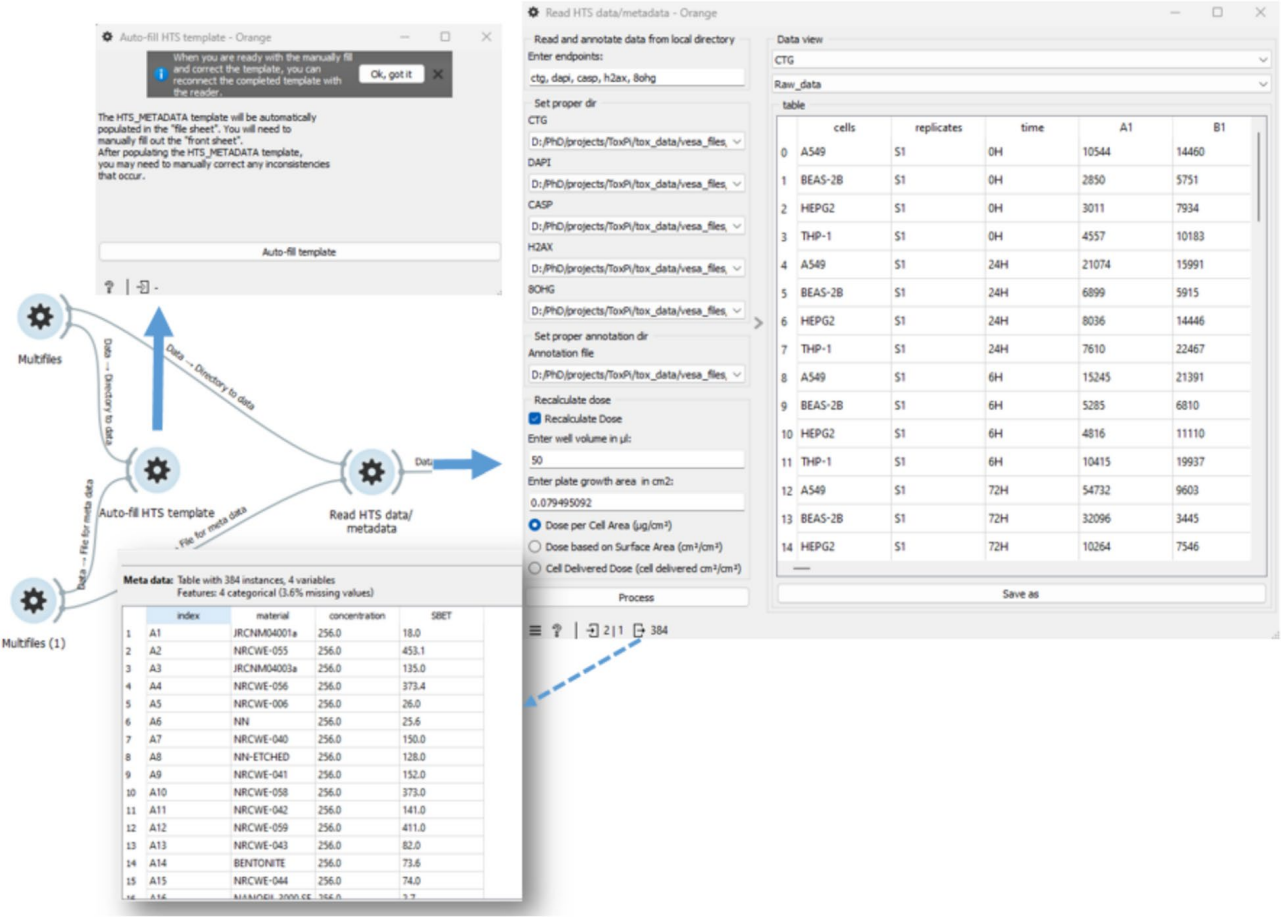
Screenshot of a specific part of a workflow, which reads and annotates HTS data

The “Multifiles” widget simplifies the file selection procedure. After selecting the directories with raw data and the harmonized metadata template, the “Auto-fill HTS template” widget (Fig. 8, top left) is used to populate the ‘file’ sheet automatically, while the rest of the sheet must be adjusted manually. Once the HTS_METADATA template is ready, it can be linked to the reader widget.

The “Read HTS data & metadata” widget features (Fig. 8, top right) are organized in four main sections: entering endpoints, selecting directories for each endpoint, setting the HTS_METADATA template, and optional dose recalculation section. After clicking “Process,” the annotated raw data is available for each endpoint, and the corresponding tables can be saved in formats like CSV or Excel. The metadata output is also available, shown in Fig. 8 with the dashed arrow.

**Fig. 9:**
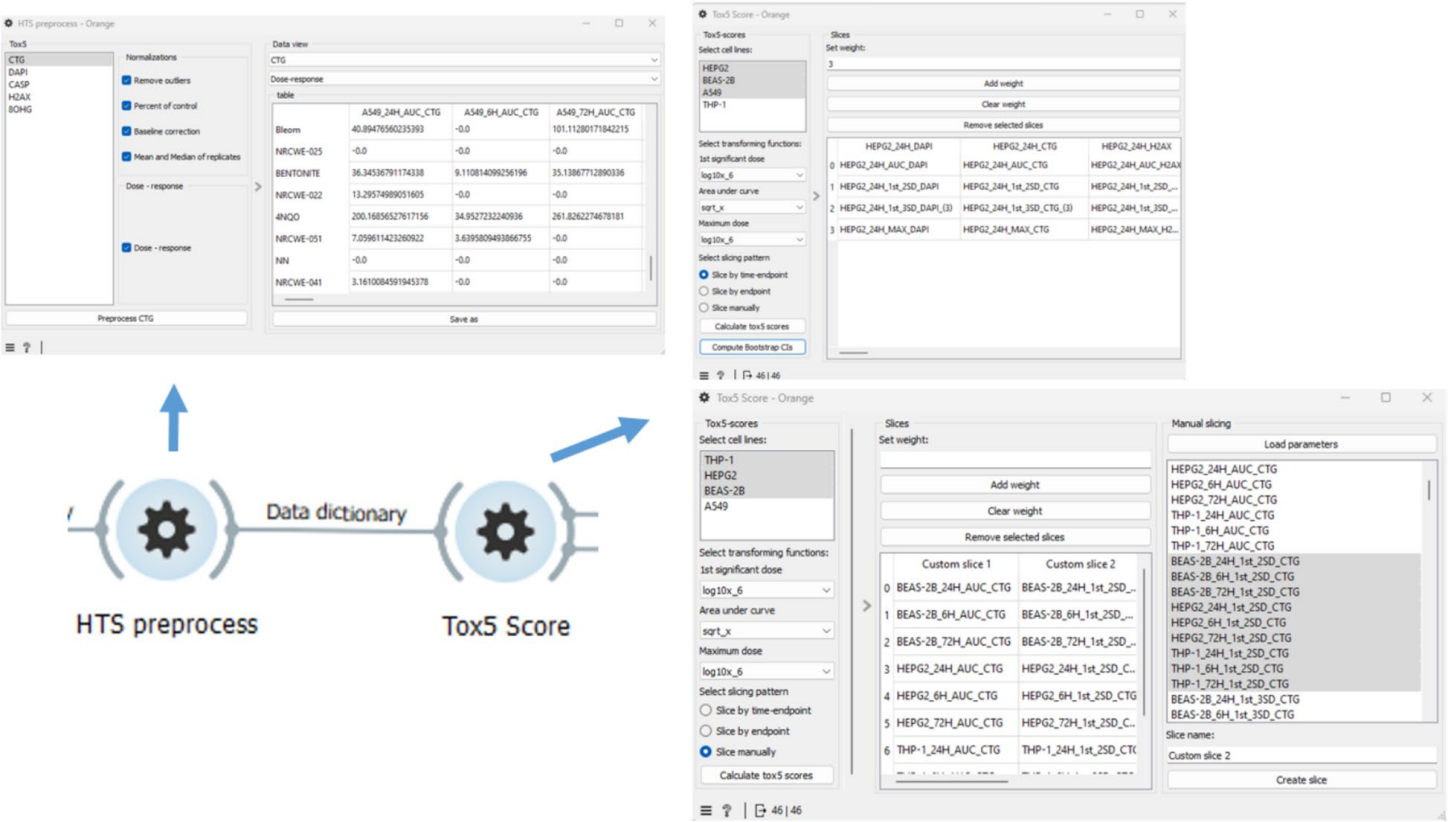
Part of an automated workflow for calculating dose–response parameters and rank materials, based on a grouping approach

The “HTS Preprocess” widget (Fig. 9, top left) implements data preprocessing and dose–response metric calculation. After selecting an endpoint, a list of normalization functions appears as clickable buttons, and the chosen functions could be applied independently to each endpoint. Results are displayed in a drop-down menu in table format. The widget takes the dictionary of defined endpoints from the previous output and returns the same dictionary after preprocessing.

The “Tox5-score” widget enables grouping and toxicity scoring, with a filter for multiple cell lines. Implemented on top of ToxPi software, it allows automatic slicing by time point and endpoint (Fig. 9 up right), with customizable slicing options (Fig. 9 down right). Users can assign weights to the metrics via a menu with options, and transform the data to a normal distribution for each dose–response metric. Bootstrap confidence intervals are also calculated as on option.

**Fig. 10:**
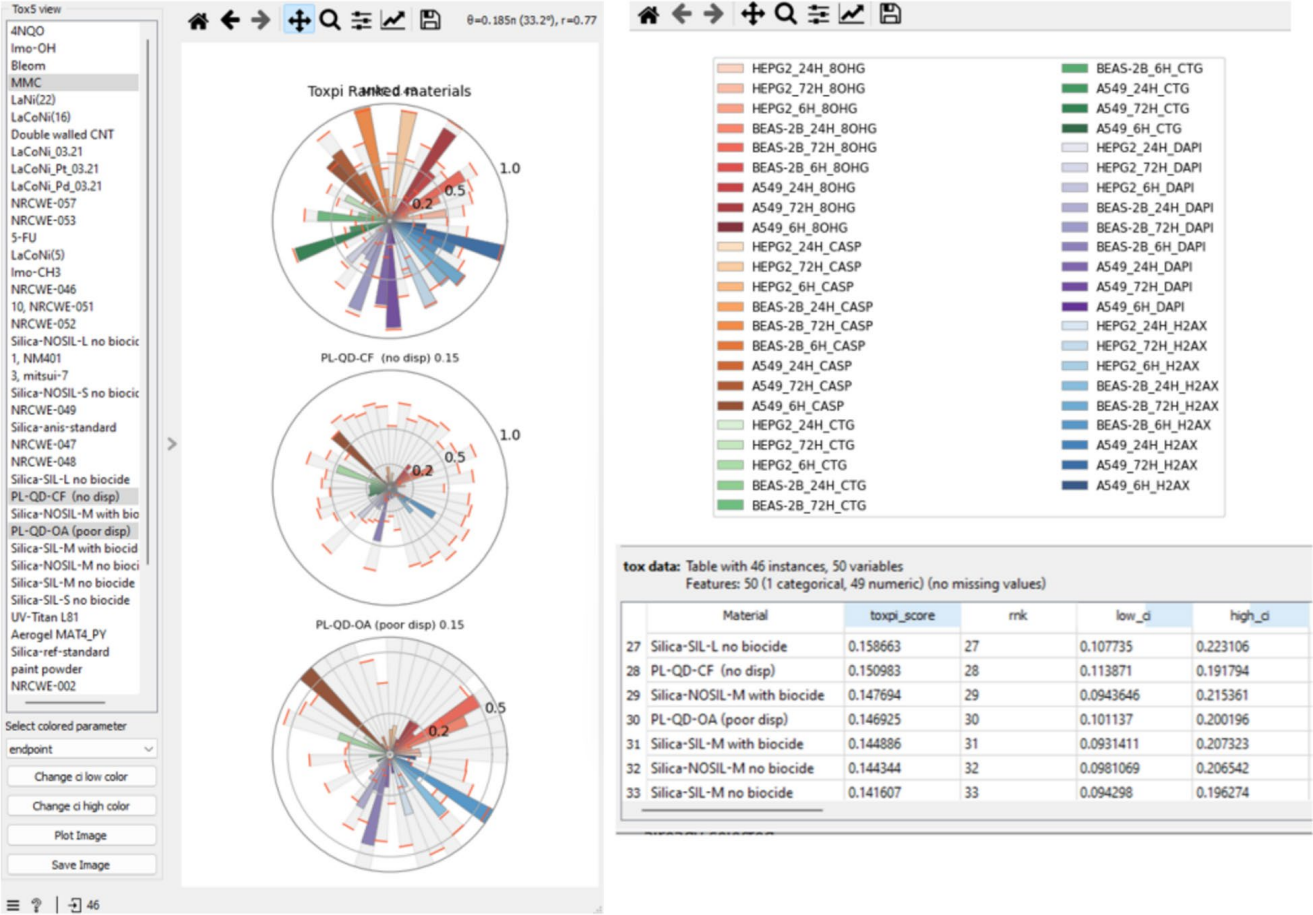
Visualization and exploration of Tox5-scores as a pie for each material

The “Tox5 view” widget (Fig. 10) displays the toxicity profiles as pies (following ToxPi approach) and materials ranking. Users can select materials from the menu on the left, export the graphic in various formats, and view the confidence intervals in gray color for each slice. An automated coloring system with custom color schemes for slices is implemented. In the example (Fig. 10), slices are colored by endpoints and organized in the legend.

**Fig. 11:**
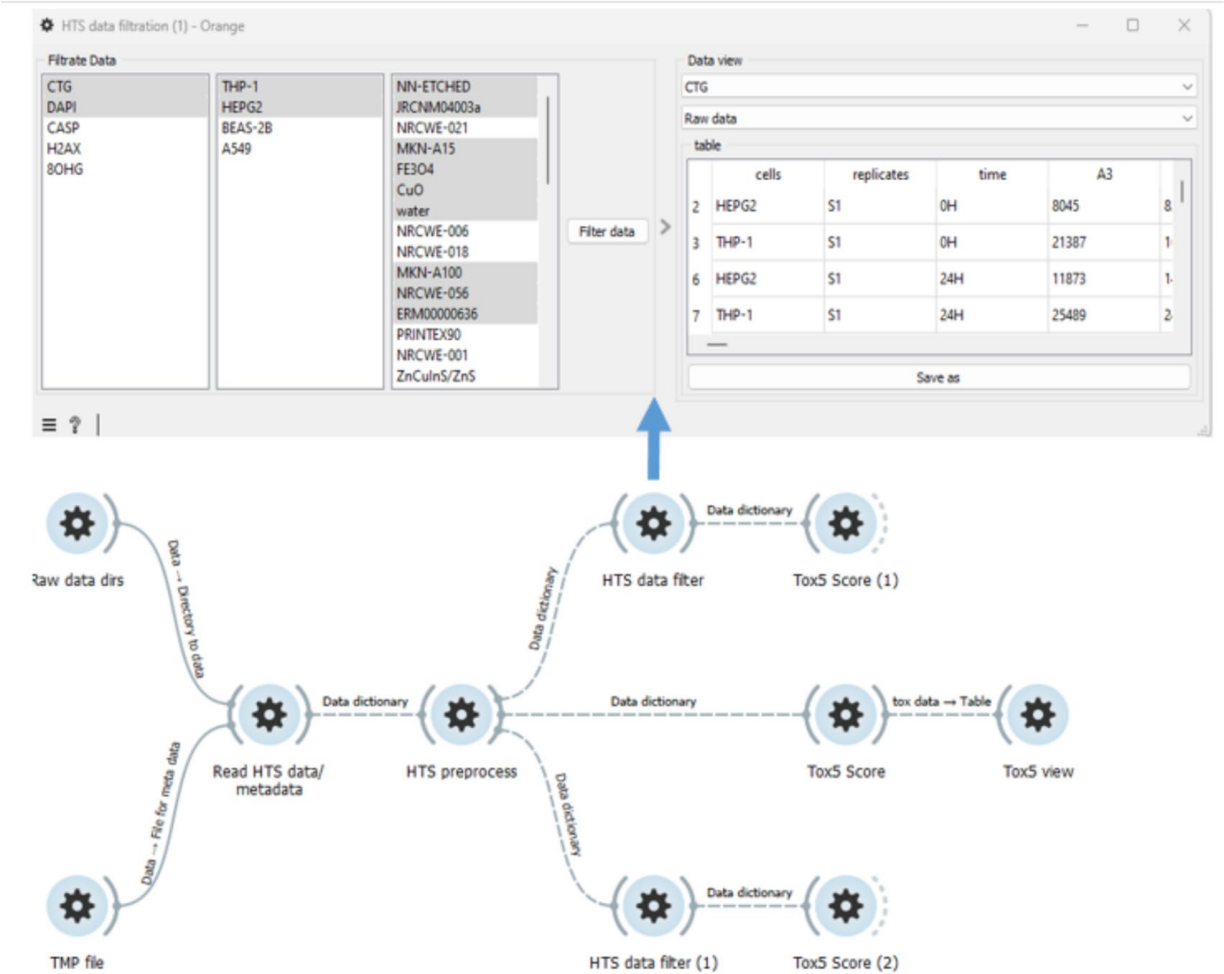
Screenshot of a workflow with different filtering use cases

The “HTS data filter” widget allows filtering of data records by endpoint, cell line and materials. It can be used before data normalization for faster preprocessing or afterwards to streamline workflows with different filters and data groupings. Figure 11 shows workflow branched for scoring behavior analysis with the same processing and corresponding slicing applied.

### Application of the ToxFAIRy workflow to the caLIBRAte dataset and HARMLESS quantum dots

#### Automatic workflow for data processing and Tox5-scoring

Experimental data for the CTG endpoint from the caLIBRAte project were retrieved from the eNanoMapper database, while all other data were obtained from a local repository and annotated using the HTS_METADATA template. Processed datasets are available as a resource in Zenodo [24].

**Fig. 12:**
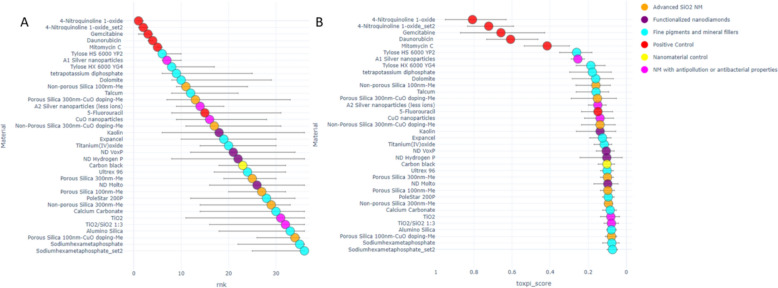
Ranked caLIBRAte materials and controls with bootstrap confidence interval, **a** by ranks and **b** by Tox5-score

The results of the automatic processing and ranking of the caLIBRAte dataset, are shown in Fig. 12. A bootstrap confidence interval is calculated for each Tox5-score (Fig. 12B) and rank (Fig. 12A), and plotted on the figure as error bars.

Different colors were used to represent the various substance types:

- Advanced SiO2 NMs (orange)—Porous Silica 300 nm-Me, Non-porous Silica 300 nm-Me, Porous Silica 100 nm-Me, Non-porous Silica 100 nm-Me, Porous Silica 300 nm-CuO doping-Me, Porous Silica 100 nm-CuO doping-Me and Non-Porous Silica 300 nm-CuO doping-Me;
- NMs with antipollution or antibacterial properties (light purple)—TiO_2_, TiO_2_/SiO_2_ 1:3, A1 Silver nanoparticles, A2 Silver nanoparticles (less ions) and CuO nanoparticles;
- Fine pigments and mineral fillers (blue)—Expancel, Titanium(IV)oxide, Dolomite, Talcum, Ultrex 96, PoleStar 200P, Calcium Carbonate, Alumino Silica, Sodium Hexametaphosphate/Sodium Hexametaphosphate set2, Tylose HS 6000 YP2, Tylose HX 6000 YG4 and tetrapotassium diphosphate;
- Functionalized nanodiamonds (dark purple)—Kaolin (Halloysite), NanoDiamond Hydrogen P, NanoDiamond Molto and NanoDiamond VoxP;
- Nanomaterial control (yellow)—Carbon black (Printex 90);
- Positive controls (red)—5-Fluorouracil, Mitomycin C, Daunorubicin, Gemcitabine, 4-Nitroquinoline 1-oxide/4-Nitroquinoline 1-oxide set2.

**Fig. 13:**
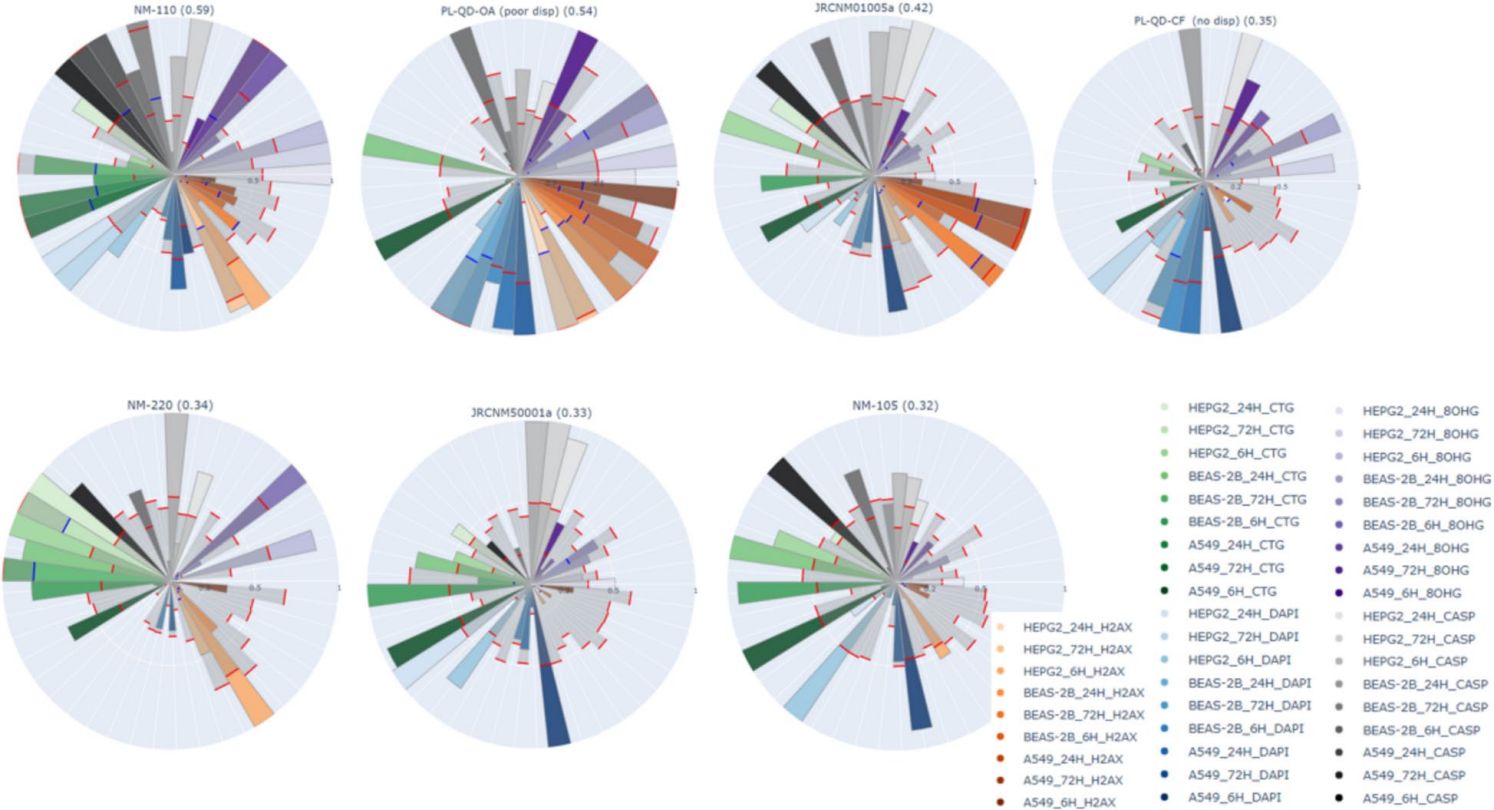
Pie chart representation of ranked Quantum dots with Tox5-scores and bootstrap confidence intervals for specific slices

Figure 13 presents the ranked quantum dots and associated reference NMs, as a pie chart, where each pie represents the entire Tox5 score for each material. Each slice corresponds to a specific score for each parameter included in the grouping and is colored according to the endpoint. For example, the CTG endpoint parameters are colored green, with a gradient indicating the specific cell lines and time points. Additionally, the bootstrap confidence interval for each slice is depicted, with the upper bound shown in red and the lower bound in blue.

**Fig. 14:**
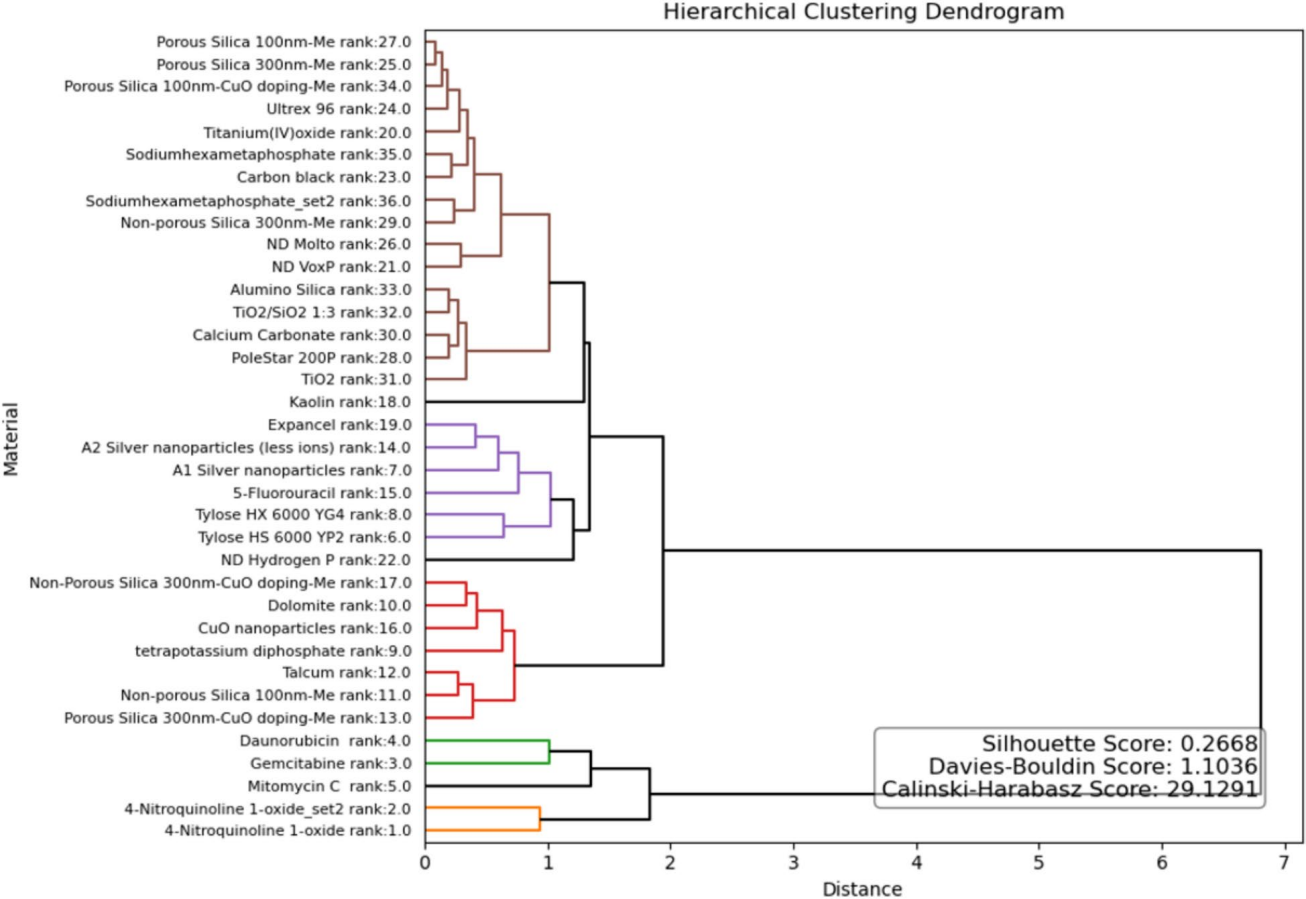
Hierarchical clustering for caLIBRAte dataset

Figure 14 illustrates hierarchical clustering applied to the caLIBRAte data, using Euclidean distance as the metric and Ward linkage method. The optimal number of clusters was determined using the “elbow” method. The “elbow” method demonstrated better statistical performance in terms of cluster significance metrics (Silhouette, Davies–Bouldin and Calinski–Harabasz scores), compared to the Silhouette method.

**Fig. 15:**
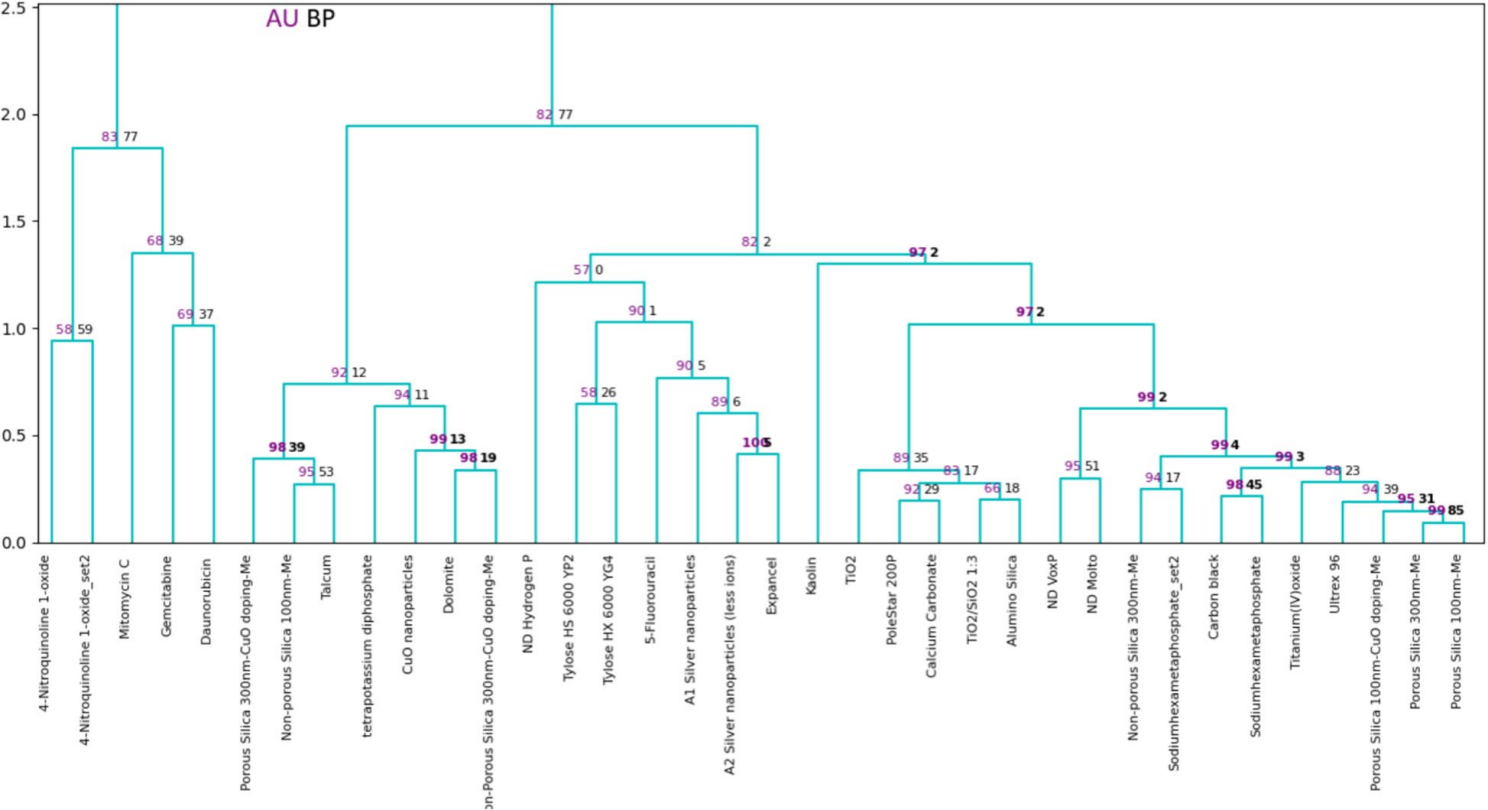
Hierarchical clustering of caLIBRAte dataset with p-values (AU/BP %) via multiscale bootstrapping

Figure 15 presents multiscale bootstrap resampling to the hierarchical clustering of caLIBRATE dataset. The clustering was done by reintegrating ‘pvclust’ [25] package in python. Approximately Unbiased p-value (AU) and Bootstrap Probability (BP) report the significance of each cluster in clustering structure. The AU value is less biased and clusters that have this value greater than 95% are considered significant and strongly supported by the data.

**Fig. 16:**
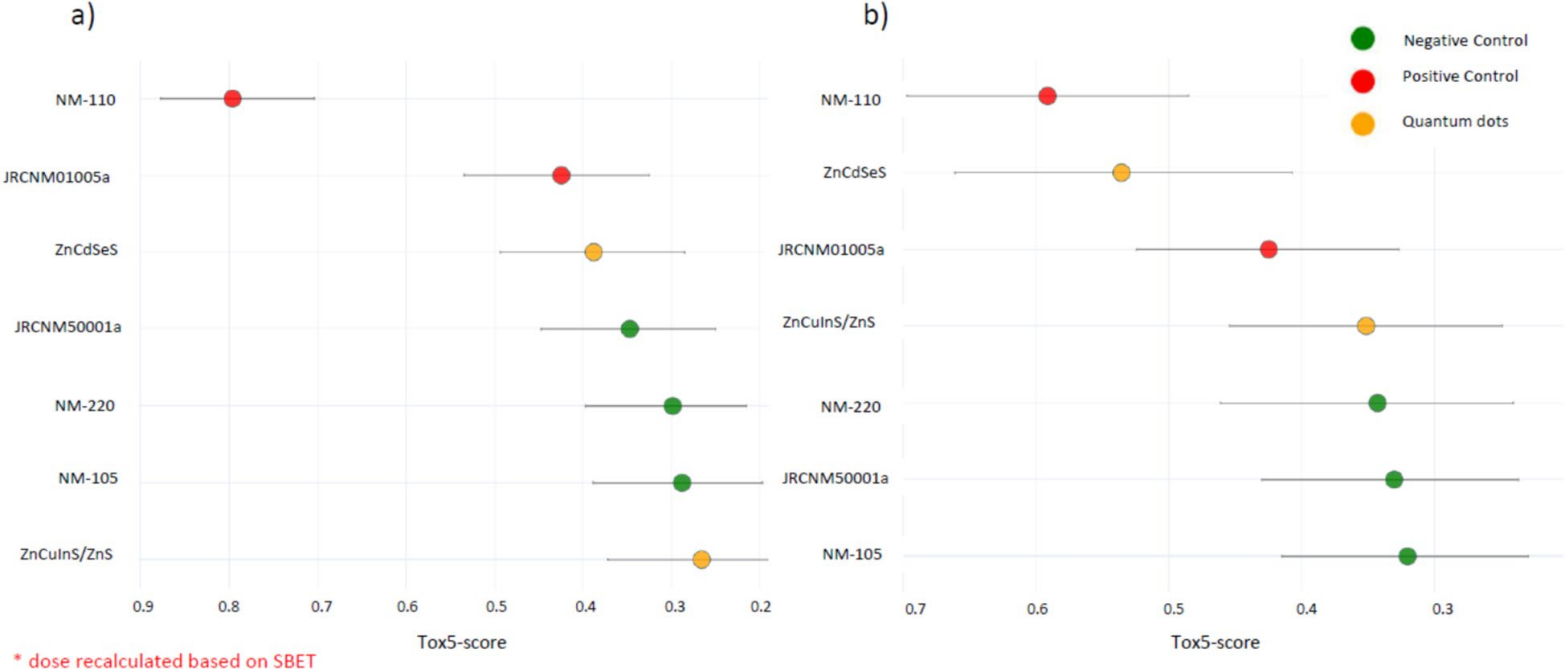
Comparison of quantum dots ranking based on recalculated doses

The HARMLESS project HTS dataset was used to test the functionality with SBET-based dose recalculations. Figure 16 demonstrates ranked quantum dots and materials used as positive and negative controls, with doses converted to cm^2^/cm^2^ (a) versus doses in µg/mL (b).

#### Biological results

Figure 12 shows ranks (A) and as Tox5 scores (B) for the caLIBRAte materials scored using both with and without serum datasets. The inclusion of positive and nanomaterial controls services as a reference for assessing the relative toxicity of the materials. Tylose HX 6000 YG4 chemically modified hydroxyethyl cellulose and TiO2/SiO2 are ranked as the most and least toxic test-material, respectively.

Toxicity effect pie charts (Fig. 13) show cell line-specific and dynamic differences in toxicity patterns between the HARMLESS quantum dots (QD) and the corresponding reference/control materials, from the PATROLS [26] project QDs decrease cell numbers (blue slices) and induce nucleic acid oxidative stress (purple slices), whereas zinc oxide (NM-110) is more prone to induce apoptosis (gray slices) and loss of cell viability (green slices).

Hierarchical clustering of the caLIBRAte materials enables grouping and read-across based on similar bioactivity, i.e. toxic modes of action (Figs. 14 and 15). The ToxFAIRy module implements bootstrap validation to identify statistically significant clusters (Fig. 15). Despite the small and heterogeneous dataset, statistically significant clusters are formed, for example, between Nanodiamonds (VoxP and Molto) and porous silica particles. As shown in Fig. 16, changing the dose metrics used can have significant effects on the toxicity ranks of nanomaterials as the specific surface areas of the materials vary dramatically. Optimally, cell-delivered doses, which take into account crucial physical–chemical characteristics such as surface areas, sedimentation rates, leaching and dissolution/ion-shedding abilities should be used for toxicity assessment.

#### High throughput data FAIRification

**Fig. 17:**
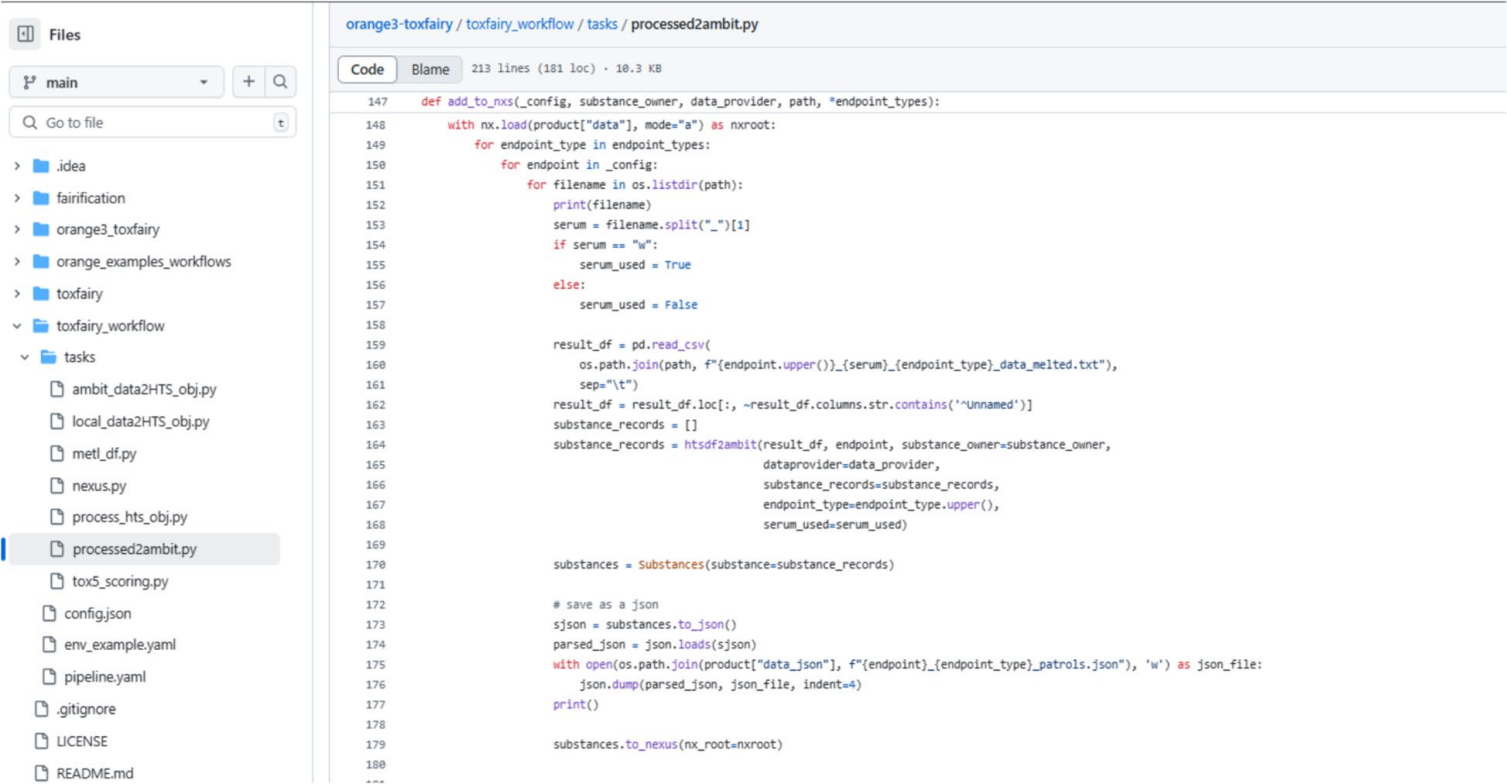
Screenshot of the code used to convert HTS data to NeXus format

The resulting data structures, after preprocessing, are converted into the FAIR eNanoMapper data model using the pynanomapper library and stored as a NeXus file. The high throughput data FAIRification is available as a widget in the Orange3-ToxFAIRy add-on and also as a specific task in a Ploomber [27] workflow. The Ploomber workflow employs the ToxFAIRy module for HTS data preprocessing, scoring and FAIRification (Fig. 17) and is available on GitHub—https://github.com/ideaconsult/orange3-toxfairy/tree/main/toxfairy_workflow.

All processed data from both datasets were converted into the FAIR eNanoMapper data model and thus made accessible for database import as well as stored in the NeXus format. The resulting NeXus file contains all HTS materials, endpoints, including raw and processed data i.e. normalized values, median values, parameters calculated from dose response curves and the Tox5-scores. The data is stored as a multidimensional matrix and H5Web tool allows interactive visualizations, selection of specific data subsets, inspection and analysis.

**Fig. 18:**
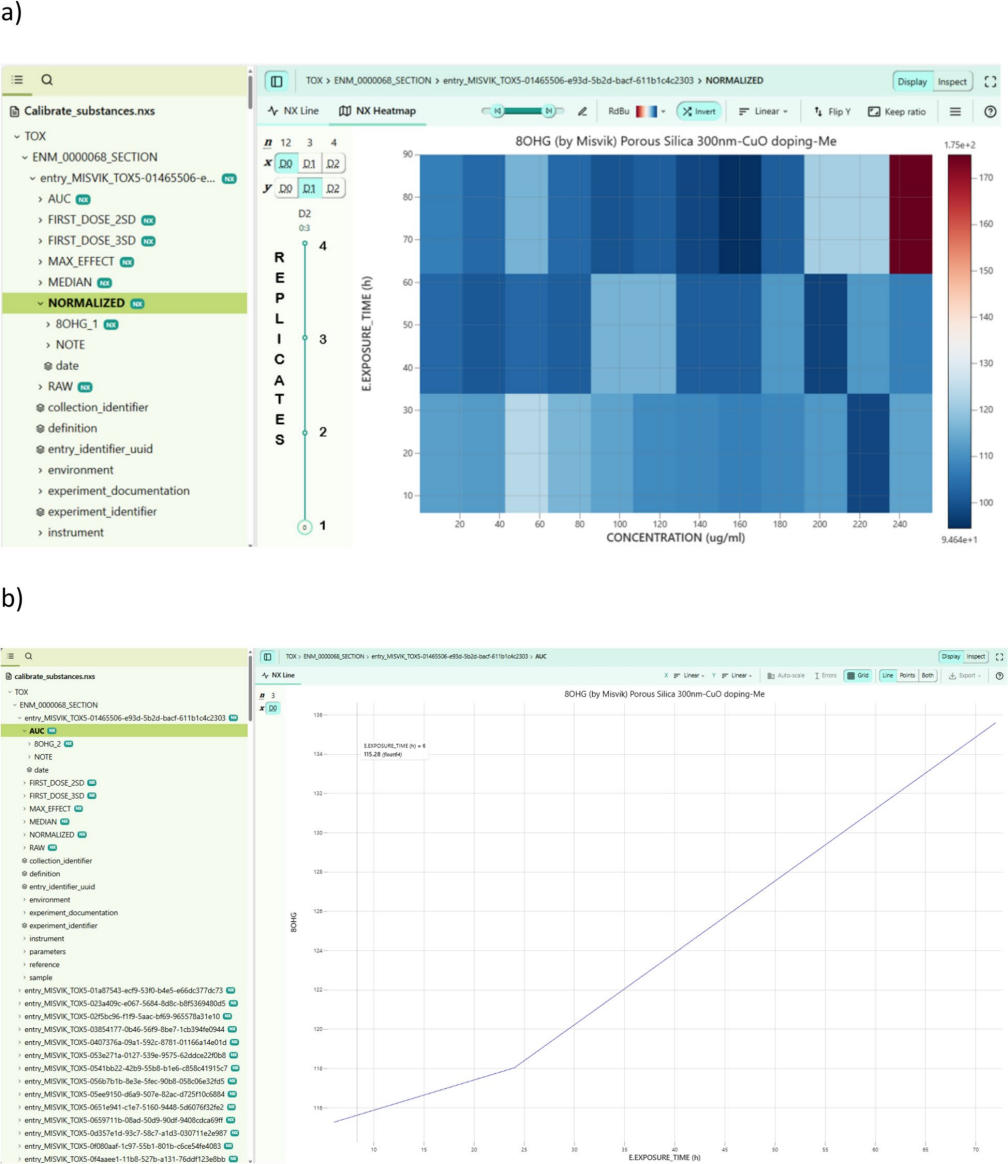
Misvik HTS data converted to NeXus format and visualized via open source h5web tool

Figure 18 contains screenshots representing the data from NeXus file with caLIBRAte datasets and displays material: Porous Silica 300 nm-CuO doping-Me, endpoint: 8OHG, cell line: BEAS-2B. Figure 18a) shows a Heatmap view for NORMALIZED data with an additional data subset of replicates in D2 and respectively, AUC results are shown in Fig. 18b).

## Discussion

The toxicological research field faces a pressing challenge in efficiently evaluating both untested existing, and novel to be developed, chemicals and materials [5],. Grouping and read-across are the recommended approaches for gap-filling for nanomaterials [28], exemplified by the hypothesis-driven Gracious framework [29] requiring information for physicochemical properties (“what they do”), fate /toxicokinetic behaviour (“where they go”) and hazard comparison based on bioactivity (“what they do”). However, linking specific physicochemical properties to well-defined hazard endpoints remains complex. To address this challenge New Approach Methodologies (NAMs) such as in-vitro testing [2, 30] or computational methods [31, 32] are increasingly being adopted, and have proved useful for assessment of bioactivity-based similarity [33].

### ToxFAIRy for automatic data processing and toxicity scoring

In vitro high-throughput screening hazard data is used for clustering, ranking, prioritization of NMs and read across. Recent efforts have led to the development of a scoring concept for evaluating and prioritizing toxicity in vitro, the Tox5-score. However, manual data preprocessing is time consuming and prone to errors, as well as unsuitable to scale-up for large NMs datasets. The original Tox5-score approach relied on manual data processing and annotation. Associated with common issues such as column shifts in Excel, and inconsistent naming of cell lines and metadata. In contrast, the ToxFAIRy automates these tasks, enhancing scalability, efficiency, and reproducibility by integrating the entire data pipeline into a unified platform. The HTS_METADATA template ensures consistent terminology and accurate metadata annotation, minimizing technical errors during data transfer.

Automation not only streamlines processing but also frees up resources for analysis, speeding up research. Key improvements over ToxPi include enhanced data visualization, easier export, automated slicing and slice coloring based on specific patterns, and the ability to add weights to specific slice parameters.

The Orange3-ToxFAIRy add-on significantly enhances user accessibility by enabling the composition of complex workflows through a visual programming interface. This visual approach simplifies the creation of workflows while enhancing their customization and flexibility. The ability to save workflows as.ows files promotes reproducibility and collaboration by allowing standardized workflows to be disseminated and reused across different research groups. Sharing these workflows ensures consistency in data preprocessing and scoring, which is crucial for reliable and comparable results.

### FAIR high throughput data retrieval and reuse

Three primary challenges are highlighted, in the context of nanomaterial grouping [29]: (a) limited data availability, (b) non-harmonized experimental methods for testing NMs, and (c) significant concerns regarding data quality, a critical issue previously noted by other researchers [34].

To overcome data scarcity, it was recommended [35] that all original data from publicly funded research projects be shared in publicly accessible databases [28]. This initiative would facilitate greater data availability, aiding in the reuse of data for purposes such as meta-analysis and NMs grouping. The eNanoMapper data model and Nanosafety Data Interface [7] is presented as an exemplary framework for creating such comprehensive data collections. The eNanoMapper data model has been effectively utilized in various EU projects and serves as a best practice [28] for data management and sharing.

Additionally, the current lack of reliable data can be addressed by implementing validated high-throughput screening approaches and predictive toxicogenomics methodologies [36, 37]. High-throughput screening platforms, which include high-content analysis and omics technologies, employed in tiered workflows and supported by computational, automated data evaluation, are crucial tools for addressing the complexity associated with NMs [35, 38, 39].

Recommendations for improving data availability are critical for acquiring the reliable data necessary to establish, enhance, and verify both NMs grouping methodologies and computational approaches in general [28]. The EU-US Nanoinformatics Roadmap 2030 [35] emphasizes the need for ongoing developments in computational nanotoxicology to support these efforts. Incorporating data into a database is crucial for efficient reuse and retrieval of the data, allowing researchers to access and leverage existing datasets for new analyses without the need to repeat experiments. This not only saves time and resources but also enhances the reproducibility of scientific studies. Ensuring that data is FAIR promotes data sharing, transparency, and long-term preservation of valuable scientific information, ultimately enhancing the overall quality and impact of research.

A significant outcome of our study is the successful extraction of FAIR HTS data from the caLIBRAte instance of eNanoMapper database, using the pynanomapper library. The retrieved FAIR HTS data have been combined with non-FAIR HTS data from the same experiment but for different endpoints. The HTS_METADATA template developed as part of the Template Wizard supports data annotation and harmonization in a reproducible manner, which is crucial for the FAIRification process.

After processing the resulting data is converted into the eNanoMapper FAIR data model and serialized in NeXus format. The main advantage of NeXus files is being machine readable, interoperable with existing HDF5/NeXus software and allow to package metadata and data of diverse experiments in the same file. These can be used for publishing in database-independent repositories like Zenodo, in electronic lab notebooks, as well as preparing an input format for data analysis.

ToxFAIRy currently supports only the pre-processing and scoring algorithms described. These approaches do not capture all relevant toxicity assessment methodologies. Future steps are to integrate additional methodologies, such as Benchmark Dose (BMD) modeling, as well as alternative scoring systems to support a tiered testing approach that incorporates physicochemical properties, ecotoxicology, and omics-based assessments.

At present, ToxFAIRy expects data from a specific source HTS format. While the HTS_METADATA template improves consistency, it may not be applicable for all types of experiments. Rather than expanding format compatibility within ToxFAIRy itself, we build upon current community efforts and open tools like Template Wizard and Template Designer and ensure that data from various sources can be converted to NeXus format. To our knowledge, this is the first proposal and software to use NeXus format for biological assays. This approach maintains interoperability while adhering to FAIR principles.

## Conclusions

We have developed an automated workflow for the FAIRification, preprocessing and scoring of high-throughput screening data (HTS) FAIRification, implemented as a new Python module, ToxFAIRy, which is accessible at Github https://github.com/ideaconsult/orange3-toxfairy. The ToxFAIRy module can be used independently or through an user friendly Orange3-ToxFAIRy add-on, enabling development of custom workflows within the Orange Data Mining platform. This integration supports visual programming for preprocessing and scoring, making the tools accessible to non-programmers.

With ToxFAIRy, the eNanoMapper FAIRification workflow can be applied to HTS data by converting the raw and processed data into the eNanoMapper semantic model, which has been previously demonstrated to enable integration of diverse nanosafety data in NanoSafety Data Interface [7], compliant with FAIR principles. Once HTS data is structured in the eNanoMapper model, ToxFAIRy offers the option to further convert it into NeXus format, a multidimensional file format that integrates raw and processed data, metadata, and hierarchical relationships into a single interoperable file.

Linking the processed data (scores) with the original raw data increases trust in the generated data and helps researchers work and analyze data more efficiently. The results of this work is a combination of methodologies for HTS data handling and newly developed software modules that can be further enhanced to include alternative scoring and ranking methods and more comprehensive modeling and data analysis. The ability to perform toxicity ranking supports implementation of safe and sustainable by design approaches by prioritizing materials with lower toxicity for inclusion in innovative processes.

In the context of the described challenges of data scarcity [28, 35], and high-throughput screening data [36, 37], and the development of computational nanotoxicology [35, 38, 39], the ToxFAIRy module contributes by harmonizing and annotating HTS data with a rich set of metadata, while minimizing manual efforts on all data analysis steps and providing conversion to an interoperable semantic data model and format. The developed tools are applicable beyond nanomaterials and HTS data and could be particularly useful for data harmonization across multiple projects and organizations and addressing the challenges with existing data processing towards integrated FAIR data resources.

## Methods

### Materials and methods for the in vitro studies

#### Nanomaterials

The 28-material selection consisted of 12 nano-components including paint additives, 8 porous and non-porous silica nanomaterials of different sizes with and without copper doping, 4 nanomaterials used as ceramic tile surface coatings, 4 functionalized nanodiamonds and Carbon black as a nanomaterial control. Five chemical controls were included in all the experiments. To test the flexibility of the automated Tox5-score workflow, 2 additional quantum dots were used from the HARMLESS project, with control materials: TiO_2_, BaSO_4_, ZnO from the PATROLS project. The treated dose could be recalculated as a μg/cm^2^ based on specific growth area and in cm^2^/cm^2^ based on SBET. The final maximum assay concentrations are indicated in Table 2.

**Table 2:** Description of materials used in the assays

Material type	Trade name (substance type)	Final max assay			BET surface m^2^/g
		ug/ml	ug/cm^2^	cm^2^/cm^2^	
Titanium(IV) oxide	Tioxide TR81	256	128		
Kaolin	Halloysite	256	128		
Nano diamond	NanoDiamond Hydrogen P	256	128		
Nano diamond	NanoDiamond Molto	256	128		
Nano diamond	NanoDiamond VoxP	256	128		
Dolomite	Microdol	256	128		
Talcum	Finntalc M15	256	128		
Calcined kaolin	Ultrex 96	256	128		
Calcium carbonate	Socal® P2 Fine Grades	256	128		
Calcined kaolin	PoleStar™ 200P	256	128		
Aluminosilicate	OpTiMat® 2550	256	128		
Expancel	Expancel 461 WE 20 d36	256	128		
Sodium-hexametaphosphate	Sodium-hexametaphosphate PLV	256	128		
2-hydroxyethyl cellulose	Tylose HX 6000 YG4	256	128		
2-hydroxyethyl cellulose	Tylose HS 6000 YP2	256	128		
Tetrapotassium diphosphate	Tetrapotassium-pyrophosphate PLV	256	128		
Silicone dioxide nanoparticle	Porous Silica 300 nm-Me (NPO_1373)	256	128	10 598	828
Silicone dioxide nanoparticle	Non-porous Silica 300 nm-Me (NPO_1373)	256	128	128	10
Silicone dioxide nanoparticle	Porous Silica 100 nm-Me (NPO_1373)	256	128	10 803	844
Silicone dioxide nanoparticle	Non-porous Silica 100 nm-Me (NPO_1373)	256	128	282	22
Silicone dioxide nanoparticle	Porous Silica 300 nm-CuO-Me (NPO_1373)	256	128	11 034	862
Silicone dioxide nanoparticle	Porous Silica 100 nm-CuO-Me (NPO_1373)	256	128	9 562	747
Silicone dioxide nanoparticle	Non-porous Silica 300 nm-CuO-Me (NPO_1373)	256	128	115	9
Copper oxide nanoparticle	CuO nanoparticles (NPO_1544)	256	128	960	75
A1 silver nanoparticles	Silver, pH 2.65	256	128		
A2 silver nanoparticles (less ions)	Silver, pH 4.56 (less Ag-ions)	256	128		
TiO_2_	TiO_2_, pH 1.01	256	128		
TiO_2_/SiO_2_	TiO_2_/SiO_2_ (1:3 ratio), pH 1.73 s	256	128		
Positive control	Gemcitabine	1.3			
Positive control	5-FluoroUracil	13.0			
Positive control	4-Nitroquinoline 1-oxide	9.5			
Positive control	Mitomycin C (CHEBI_59999)	33.4			
Positive control	Daunorubicin	10.6			
Carbon black	Printex 90/nanomaterial control	256	128		
Quantum dot	PL-QD-CF ZnCuInS core/ZnS shell	256	161	397.97	247.16
Quantum dot	PL-QD-OA ZnCdSeS no shell	256	161	237.34	147.4
Positive control	ZnO (NM-110)	256	161	17.92	14
Negative control	BaSO4 (NM-220)	256	161	52.48	41
Positive control	TiO_2_ (JRCNM01005a, NM-105)	256	161	59.11	46.175
Negative control	BaSO_4_ (JRCNM50001a, NM220)	256	161	52.48	41
Negative control	TiO2 (NM-105)	256	161	59.11	46.175

All materials from Table 2 were measured in the caLIBRAte project except quantum dots. Joint Research Centre (JRC) materials with JRC code are different batches of the same materials.

#### Cell models

The lung epithelial A549 cells were acquired from ECACC (86012804, LOT 17L047, passage + 92) and cultured in RPMI 1640 (11879020 Gibco) supplemented with 1% Penicillin–Streptomycin (15140-122 Gibco), 1% l-Glutamine (17-605E Gibco) and 10% FBS (10270-106, heat inactivated, Gibco), (500 ml RPMI + 5.5 ml Pen-Strep + 5.5 ml l-Glutamine + 50 ml FBS).

Human bronchial epithelial BEAS-2B cells (Sigma-Aldrich #95102433) were cultured without serum using LHC-9 bronchial epithelial cell growth medium (Thermo-Fisher Scientific/Gibco #12680013). The caLIBRAte screens were carried out ± 10% fetal bovine serum (Biowest S181B-500) and Harmless screens only with + serum, added to the LHC-9 medium.

Liver HepG2 cells were acquired from ECACC (85011430, LOT 17K028, passage 100) and cultured in DMEM (with 4.5 g/l d-Glucose, l-Glutamine, 41966-029 Gibco) supplemented with FBS (10270-106, Gibco) and Penicillin/Streptomycin (15140-122, Gibco), (500 ml DMEM + 50 ml FBS + 5 ml Pen-Strep).

THP-1 Monocyte cells were acquired from ECACC (#88081201) and cultured in RPMI-1640 (11879020, GIBCO®, Paisley, UK) supplemented with 1% Penicillin–Streptomycin (15140-122 Gibco), 1% l-Glutamine (17-605E Gibco) and 10% FBS (10270-106, Gibco), 0.9% HEPES buffer, 0.9% Sodium pyruvate (500 ml RPMI + 5.5 ml Pen-Strep + 5.5 ml l-Glutamine + 50 ml FBS + 5 ml Hepes + 5 ml Sodium pyruvate). THP-1 cells were differentiated into Macrophages (dTHP-1) over 48 h with 100 nM PMA without recovery phase before each screen.

The assays have been conducted using the BEAS-2B cell line, and quantum dots have been employed across all human cell types. The cells were cultured in 10 cm dishes (Thermo Scientific 130182) and the assays were carried out in 384-well plates (Greiner µ-clear plates with a 10 mm^2^ growth area per well, #781091 and Corning with a 7.95 mm^2^ growth area per well, #3771 for quantum dots.) using cells under passage 20. The cells were passaged at ~ 80% confluence using Accutase cell detachment solution (Thermo-Fisher Scientific/Invitrogen # 00-4555-56) at 1:2–1:6 ratio twice a week.

#### Nanomaterial dispersion and high throughput screening

The NANOGENOTOX SOP was used for dispersions. A calorimetric testing was carried out according to the SOP provided for Hielscher SP200-st sonicator and samples were sonicated for 9 min and 38 s at 20% amplitude using a 14 mm probe to yield 7056 Joules of acoustic energy to the samples. The materials were dispersed to yield 6–8 ml of 2.56 mg/ml solution. Following each dispersion, the samples were aliquoted to 2 ml tubes and stored at − 80 °C.

After the dispersions, the nanomaterials were thawed, thoroughly vortexed and pipetted as 12-concentration dilutions series with twofold concentration increase per step into assay master-plates (Eppendorf deep well 384 plates #0030522.109), which were sealed and stored at − 80 °C before preparation of assay plates (Greiner µ-clear 384-well plates) for which 5 µl of the nanomaterial solution per well was pipetted from the master plates using Eppendorf epMotion 96 liquid handling station. The assay plates were heat-sealed and stored at − 20 °C before the screens. The time between dispersions and the screens was kept as short as possible.

On the screening day the cells were detached from culture plates using Accutase cell detachment solution, counted using a Nexcelom bioscience Cellometer mini cell counter and dispersed (1200 cells/well in 45 µl) to 384-well assay plates containing the nanomaterials using a Multidrop 384 dispersion station (Thermo Scientific/Titertek). The cells were exposed to the nanomaterial, together with a panel of class representative chemical controls, for 6, 24 and 72 h (4-Nitroquinoline 1-oxide [4NQO, Sigma-Aldrich 442683], Fluorouracil [5-FU, Sigma-Aldrich F6627], Daunorubicin [Sigma-Aldrich D8099], Gemcitabine [Sigma-Aldrich G6423], mitomycin C [MMC, Sigma-Aldrich Y0000378]. An additional 0-h time point was used in the CTG screens as a background control plate.

The quantum dots were dispersed as it was done in the caLIBAte project, except for 90 min to yield 68137 Joules of acoustic energy to the samples due to poor dispersibility.

#### Cell viability measurement

For the cell viability measurement, the cells were lysed by adding 5 µl of the CellTiter-Glo (Promega, G7573) reagent to each well and the total ATP content was measured after 15 min using a Labrox plate reader.

#### DAPI, γH2AX, 8OHG and Caspase-3 staining

The staining for γH2AX (Cell Signaling CS9718), 8OHG (Abcam ab62623), DAPI (Sigma-Aldrich 10236276001) and Caspase-3 (Cell Signaling CS9661) was carried out in 384-well plates in the following way: the cells were fixed at the given time points in 384-well plates by removing 1/2 of the medium and by adding 25 µl of 1/5 diluted 37% formaldehyde (Sigma # 252549, 3.7% final) with Eppendorf EpMotion 96 for 15–30 min. The plates were aspirated empty, 50 µl PBS added and the plates stored sealed at + 4 °C before staining. To stain, the cells were permeabilized with 10 µl of 0.3% Triton-PBS for 10–30 min, washed twice with PBS, 7 µl per well of primary antibody in 2% BSA-PBS was added (Anti-rabbit µH2AX 1:600, Anti-mouse 8OHG 1:600, Anti-rabbit Caspase-3 1:600), the plates spun down and kept overnight in dark at 4 °C. The next day the plates were washed twice with PBS, 7 µl of secondary antibody per well with DAPI (1:1000) added in 2% BSA-PBS, spun down and incubated in dark for 60 min [Donkey anti-rabbit 568-Alexa 1:425 (A10042), Donkey anti-mouse 647-Alexa 1:425 (A31571)]. The plates were then washed twice with PBS, left in PBS, sealed, and stored at 4 °C before imaging. The 8OHG antibody recognizes both RNA and DNA which are in the cytoplasm following oxidative stress. The 8OHG staining was almost exclusively cytoplasmic.

#### Image analysis

Automated microscopic analysis of cells was carried out with high-content imaging station ScanR (Olympus) equipped with inverted microscope IX81 and Hamamatsu ORCA-ER high sensitivity cooled CCD-camera (Hamamatsu Photonics K. K.). Four images were acquired with 10× magnification using specific filter sets for DAPI, Alexa568 and Alexa647 (Invitrogen) labels. The software automatically removes the background image. Cells were first segmented based on DNA counterstaining (DAPI) by edge-finding algorithm and the average nuclear fluorescence intensities of DAPI and Alexa568 were measured (only nuclear γH2AX was measured). Average cytosolic fluorescence intensity was recorded from a predefined area outside of the nucleus (cytoplasmic and nuclear 8OHG and Caspase-3 was measured). Clustered cell populations with high or low fluorescent intensity of Alexa568 were gated from two-dimensional scatter plots (Nuclear DAPI intensity vs. area). Nanomaterial induced artifacts were removed by circularity factor vs. elongation factor gating before the cell number (DAPI) assessment. The well median intensity values were extracted and values two standard deviations above the median of control values were counted as µH2AX, 8OHG and Caspase-3 staining positive.

The quantum dots HTS screens were carried out as eight-concentration dilutions series with threefold concentration increase per step. Thermo Scientific™ Abgene™ 96 deepwell plates (AB-0661) were used as master plates and 20 µl (5 µl of NM + 15 µl of serum free medium) of the NMs solution per well was pipetted from the master plates into the assay plates. On the day of the screening the cells (950 cells in 30 µl of medium = the same final cell density as in caLIBRAte) were added to 384-well assay plates containing the NMs. For the apoptosis assays, supernatants were drained from the wells using a 16-channel manifold, 10 µl of 50:50 Caspase-Glo 3/7 (Promega, G8093)—PBS added to the wells and the signal measured after 45 min. To control for assay interference, an additional 0-h apoptosis time point was introduced and the background signal was measured by adding 20 µl of undiluted Caspase-Glo to the wells.

### Materials and methods for used software and data formats

#### Toxicological prioritization index (ToxPi) software

The ToxPi [18] is an analytical framework that was developed to enable integration of multiple sources of evidence by transforming data into integrated, visual profiles. For each ToxPi material, a dimensionless index score ranging from 0 to 1 is calculated as a weighted combination of all data sources that represents a formalized, rational integration of information from different domains. ToxPi represents this integration visually as component slices of a unit circle, with each slice representing one piece of information. For each slice, the distance from the origin (center) is proportional to the normalized value of the data points for the component comprising that slice, and the width (in radians) indicates the relative weight of that slice in the overall calculation of ToxPi.

#### Orange data mining software

The orange data mining system [40, 41] is an open-source visual programming tool for data analysis and mining, supporting macOS, Windows, and Linux. It has a user-friendly drag-and-drop interface, allowing even non-programmers to build data workflows by connecting widgets for tasks like data import, transformation, visualization and machine learning. Orange supports various data formats and offers pre-processing tools for cleaning and manipulating data. It includes a range of machine learning algorithms and visualization options for easy exploration. Users can create custom widgets in Python. We utilized the Orange interface to implement our Python module, and we developed custom widgets that are available as a new Orange add-on.

### Data FAIRification and the importance of the semantic data model

The FAIR Principles for Scientific Data Management [6] aim to enhance the discoverability, accessibility, interoperability, and reusability of data. Emphasizing machine actionability, these domain-agnostic principles address the growing reliance on computational support in research. GO-FAIR [42] defines FAIRification as a transformative process that begins with data acquisition, analysis, and the definition of a semantic model. The subsequent steps include making data linkable, assigning a well-defined license, defining metadata, and deploying FAIR data for machine readability and linkage with other sources.

While terminology alignment is a common step, the most resource-intensive phase in the FAIRification process is defining the semantic model, or aligning the data to it. A semantic data model comprises objects representing aspects of reality and their relationships. Researchers routinely describe the primary data objects for experiment description in scientific literature as: materials, methods and results. Computer representation of the experimental system as domain data structure and enabling machine actionality require defining the data objects, as well as the relationships and constraints between them. In computer science, this is known as a data model, and serves as a blueprint for how data will be stored, accessed and manipulated. Conceptually, a data model is different from a data format, as one and the same data model can be stored in different formats.

The main objects in the eNanoMapper data model [7] are material(s), methods, the measurement processes (the specific assays utilized to generate the end-point data) and the experimental results.

#### NeXus data format

NeXus data format [43, 44] is a community designed format to accommodate complex and diverse datasets based on Hierarchical Data Format version 5 (HDF5), a widely used open scientific format optimized for sets of data matrices organized in a hierarchical structure. The “measurement process” entity from the eNanoMapper data model corresponds to the NeXus “NXEntry” object and includes all objects starting with “entry_”. NXEntry is the main container for experimental data, while NXProcess represents processed data. The results can be scalar values, array of values or multi-dimensional matrices (e.g. spectra, images, dose response).

NeXus files are structured with predefined classes like NXentry, NXdata, and NXsample, ensuring consistent data organization. NXdata groups hold experimental data, including scalar values or multi-dimensional arrays, along with associated metadata such as units, error information and axes. NeXus supports linking data fields to establish relationships between datasets.

NeXus format is appropriate for storing annotated datasets from multiple experimental techniques, hence the pynanomapper library https://github.com/ideaconsult/pynanomapper/ have been updated with the ability to convert the eNanoMapper data model into NeXus data format. The NeXus format enables to package multiple types of experiments into the same file. The processed data can be stored under NXProcess group instead of NXentry group. Derived values, e.g. normalization, mean, dose–response parameters and Tox5-scores, can be stored in separate datasets. Most of the eNanoMapper/AMBIT data model components (entities) have clear correspondence to NeXus data structures and vice versa.

Visualization of the NeXus format is enabled by ready-to-use open-source tools developed by the scientific community, namely H5Web [45] and NexPy [45], and online supplied tools (e.g. myHDF5 [46]).

## Data Availability

The datasets supporting the conclusions of this article are available in the Zenodo repository: 10.5281/zenodo.13683162. The ToxFAIRy software and Orange3-ToxFAIRy Orange add-on are available online with MIT license at GitHub repository: https://github.com/ideaconsult/orange3-toxfairy. The visual guide for Orange3-ToxFAIRy add-on is also available in the Zenodo repository: 10.5281/zenodo.13685297. Data entry template for HTS data, with links to data files is available in Zenodo repository: 10.5281/zenodo.14260137.

## Abbreviations

HTS: High-throughput screening
NMs: Nanomaterials
NAMs: New Approach Methodologies
ECHA: European Chemicals Agency
EPA: Environmental Protection Agency (USA)
TSCA: Toxic Substances Control Act (USA)
FAIR: Findable, Accessible, Interoperable, and Reusable
ToxPi: Toxicological Prioritization Index
BET: Brunauer–Emmett–Teller method
SBET: Specific Surface Area measured by BET method
NaN: Not a Number
AUC: Area under the curve
JSON: JavaScript Object Notation
SOP: Standard operating protocol
DOI: Digital Object Identifier
JRC: Joint Research Centre
HDF5: Hierarchical Data Format version 5
CI: Confidence interval
AU: Approximately unbiased
BP: Bootstrap probability
QD: Quantum dots
DAPI: 4ʹ,6-Diamidino-2-phenylindole
